# Structural transitions upon guide RNA binding and their importance in Cas12g-mediated RNA cleavage

**DOI:** 10.1371/journal.pgen.1010930

**Published:** 2023-09-20

**Authors:** Mengxi Liu, Zekai Li, Jing Chen, Jinying Lin, Qiuhua Lu, Yangmiao Ye, Hongmin Zhang, Bo Zhang, Songying Ouyang

**Affiliations:** 1 Key Laboratory of Microbial Pathogenesis and Interventions-Fujian Province University, the Key Laboratory of Innate Immune Biology of Fujian Province, Biomedical Research Center of South China, Key Laboratory of OptoElectronic Science and Technology for Medicine of the Ministry of Education, College of Life Sciences, Fujian Normal University, Fuzhou, China; 2 Department of Biology, Southern University of Science and Technology, Shenzhen, Guangdong, China; Cornell University, UNITED STATES

## Abstract

Cas12g is an endonuclease belonging to the type V RNA-guided CRISPR–Cas family. It is known for its ability to cleave RNA substrates using a conserved endonuclease active site located in the RuvC domain. In this study, we determined the crystal structure of apo-Cas12g, the cryo-EM structure of the Cas12g-sgRNA binary complex and investigated conformational changes that occur during the transition from the apo state to the Cas12g-sgRNA binary complex. The conserved zinc finger motifs in Cas12g undergo an ordered-to-disordered transition from the apo to the sgRNA-bound state and their mutations negatively impact on target RNA cleavage. Moreover, we identified a lid motif in the RuvC domain that undergoes transformation from a helix to loop to regulate the access to the RuvC active site and subsequent cleavage of the RNA substrate. Overall, our study provides valuable insights into the mechanisms by which Cas12g recognizes sgRNA and the conformational changes it undergoes from sgRNA binding to the activation of the RNase active site, thereby laying a foundation for the potential repurposing of Cas12g as a tool for RNA-editing.

## Author summary

Cas12g is an RNA-guided ribonuclease (RNase) that displays both collateral RNase and single-stranded DNase activities distinguishing it from other Cas12 proteins discovered so far that targeting DNA. RNA-targeting CRISPR-Cas effectors have been developed into various tools for manipulating specific RNA molecules. Currently, RNA-targeting applications primarily rely on the RNA-specific type VI systems, particularly Cas13a-13d, which possess HEPN domains that are exclusively involved in RNA cleavage. Although both Cas12g and Cas13 effector proteins possess the ability to cleave RNA, they exhibit significantly different characteristics in terms of structure and molecular mechanisms. To harness Cas12g and apply it as an effective RNA editing tool, it is important to understand the molecular basis for sgRNA recognition as well as target RNA recognition and cleavage by Cas12g. Our findings not only improve understanding of Cas12g at molecular level but also provide useful information for rational engineering of the CRISPR-Cas12g system and its application in RNA editing and other fields.

## Introduction

CRISPR–Cas is part of the defense systems of the adaptive immunity of bacteria and archaea against bacteriophages and mobile genetic elements. It uses Cas effector proteins to target and cleave exogenous nucleic acid sequences that match the guide RNA [1–4]. Class 2 CRISPR–Cas systems, such as CRISPR–Cas9, CRISPR–Cas12, CRISPR–Cas13, consist of single multidomain Cas effector proteins compared to the multisubunit effector complexes found in class 1 CRISPR–Cas systems. These class 2 systems have been effectively applied in genome editing and molecular diagnostics [5–11]. To further expand the CRISPR–Cas toolbox, recent research has focused on identifying and studying new subtypes of class 2 systems, particularly type V systems [12,13]. The recently identified type V effector proteins, including Cas12c [14,15], Cas12i [16,17], and Cas12f [18,19], have been discovered to possess RNA-guided double-stranded DNA (dsDNA) interference activity, similar to the classic Cas12a and Cas12b proteins. Thus far, the unifying feature of type V effectors is the presence of a conserved RuvC-like endonuclease domain. However, while most known type V effectors use this domain to cleave DNA substrates, Cas12g, the Cas effector of the newly identified type V-G systems, stands out by exhibiting RNA-guided ribonuclease (RNase) activity instead of cleaving DNA substrates [12].

Cas12g, which is a thermostable protein identified from a hot spring metagenome locus, functions as an RNA-guided ribonuclease (RNase) [12]. It possesses both collateral RNase and single-stranded DNase activities. Cas12g also exhibits unique characteristics that differentiate it from other Cas12 effectors, such as: 1) it does not rely on a protospacer adjacent motif (PAM) for target recognition, 2) it is guided by a mature CRISPR RNA (crRNA) hybridized with trans-activating crRNA (tracrRNA) or the covalently fused single-guide RNA (sgRNA), and 3) Cas12g is unable to process its precursor crRNA (pre-crRNA) *in vitro* with or without tracrRNA, suggesting that additional endogenous factors are required for *in vivo* crRNA biogenesis [12]. Moreover, the relatively small size of Cas12g (767 amino acids [aa]) makes it suitable for packaging into therapeutic viral vectors such as adeno-associated virus (AAV) and for application in transcriptome engineering. To harness Cas12g as an effective tool, it is important to comprehend its molecular basis for sgRNA recognition, as well as its target RNA recognition and cleavage.

Recently, the cryo-EM structures of Cas12g bound to sgRNA and sgRNA-target RNA have been published [20]. Although the Cas12g-sgRNA complex has been extensively characterized, the region spanning aa 220–354 in the Helical 1, which plays a crucial regulatory role in target RNA recognition and Cas12g activation, is poorly resolved in the cryo-EM map. Additionally, the structural element of the lid motif located in the RuvC domain is conserved in Cas12a [21], Cas12b [22], Cas12i [16] and Cas12j [23] and can sense the formation of the crRNA-target DNA duplex. The lid motif of Cas12g has been found to be vital for RuvC activation [20]. However, the RuvC activation regulated by the lid motif in Cas12g needs further investigation. Thus, further investigation is required to understand RNase activation and the mechanism of target RNA cleavage.

To address these questions, we present the structures of two different states: apo-Cas12g and the Cas12g-sgRNA binary complex. The structural analysis of these two states provides insight into the recognition and interaction of Cas12g with sgRNA. Upon binding to sgRNA, we found that Cas12g undergoes conformational rearrangements, creating a positively charged central channel that accommodates sgRNA. Notably, the conformational helix-to-loop transition of the lid motif in RuvC induced by sgRNA binding leads to partial exposure of the RuvC active site, preparing it for substrate binding and cleavage. Additionally, in the apo-Cas12g structure, we identified two adjacent zinc finger motifs within the Helical 1 subdomain II (aa 201–382). Overall, this study confirms the significant role of these zinc finger motifs in the substrate cleavage process. By integrating our findings with previous structural research [20], we propose a comprehensive multistep mechanistic model for Cas12g that explains both its activation and RNA cleavage activities. This study not only enhances our understanding of the substrate cleavage mechanism of Cas12g, but also suggests a new approach for developing RNA editing tools by leveraging the interplay between structure and function.

## Results

### Overall structure of apo-Cas12g

**Fig 1:**
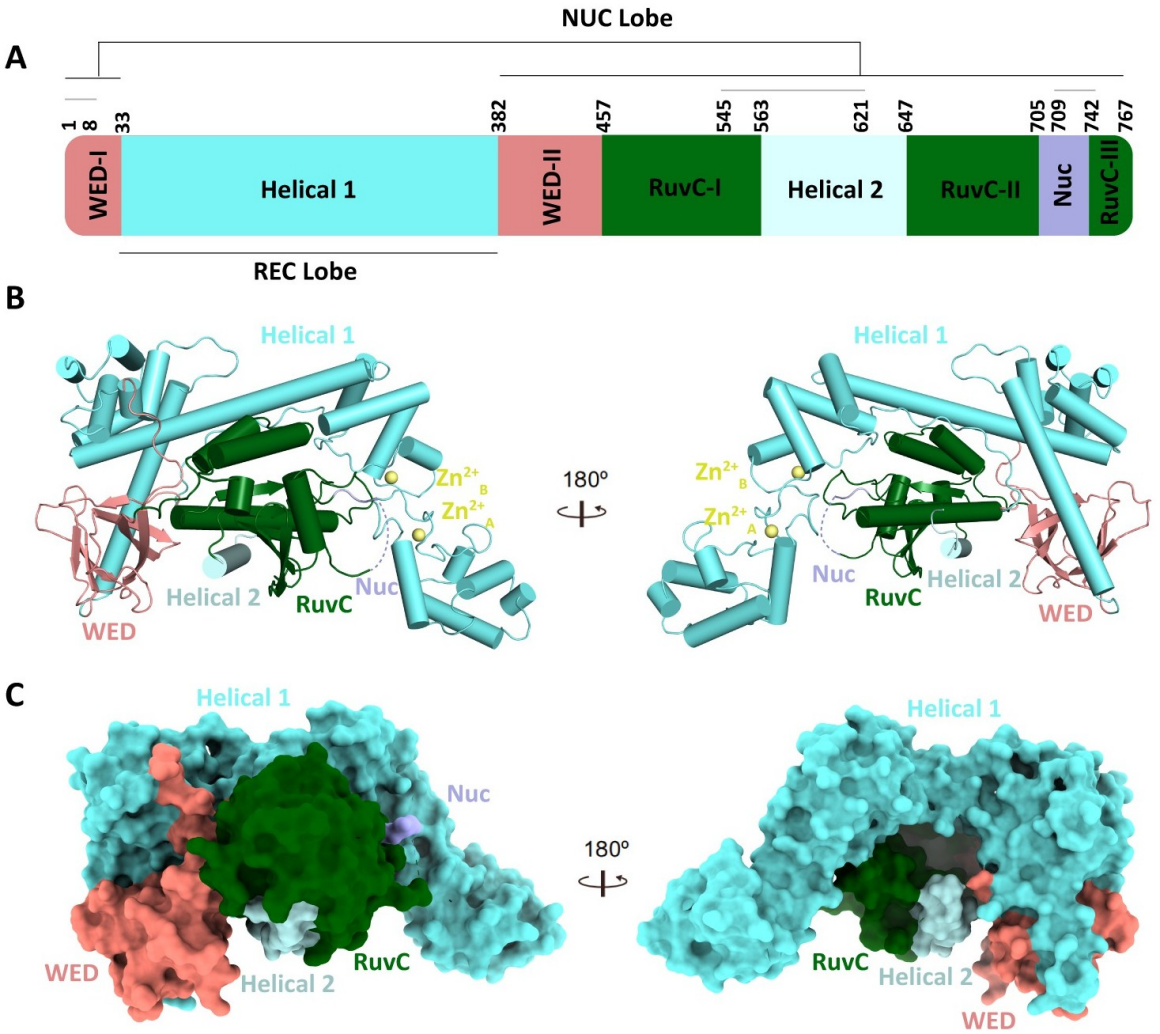
Crystal structure of apo-Cas12g. **(A)** Domain organization of Cas12g. The amino acid segment marked by the solid gray line represents the unresolved region. **(B)** Overall structure of apo Cas12g shown in one view (left) and rotated through 180° (right). **(C)** Surface representations of apo Cas12g in the same views as in **(B)**.

The crystal structure of apo-Cas12g was determined at a resolution of 2.24 Å. We observed a single Cas12g in the asymmetric unit of the crystal structure. Apo-Cas12g exhibits a compact domain arrangement that differs from the extended conformations observed in apo-Cas12a [24]. Cas12g adopts a bilobed architecture encompassing recognition (REC) and nuclease (NUC) lobes (Fig 1). The REC lobe solely contains the Helical 1 domain, while the NUC lobe consists of WED, Helical 2, RuvC and Nuc domains (Fig 1A). The Helical 1 domain consists of two subdomains: subdomain I (aa 34–200) and subdomain II (aa 201–382). The Helical 1 subdomain I forms the hinge-proximal region of the REC lobe and consists of helices α1–α8. Comparatively, the Helical 1 subdomain II forms the hinge-distal region of the REC lobe and consists of helices α9–α17 (S3C Fig). Notably, two zinc finger motifs, HCCC-type and CCHC-type, were identified in the Helical 1 subdomain II (Fig 1B). Cas12g lacks a PAM-interacting domain, which is consistent with the observation that a PAM sequence is not required for target recognition [12].

The WED domain is divided into WED-I and WED-II subdomains, which can fold into two antiparallel β-sheets (β1-β2-β6-β7 and β3-β4-β5) and act as a hinge connecting the REC and NUC lobes (S3A and S3F Fig). The RuvC domain, essential for RNase activity, can be divided into RuvC-I, RuvC-II and RuvC-III subdomains, adopting a conserved RNase H fold in which the β-sheet is surrounded by five α-helices (S3G Fig). The Helical 2 domain (aa 563–647) is sandwiched between RuvC-I and RuvC-II. However, the density for the segment spanning aa 545–621 is absent, and only a helix can be observed in the Helical 2 domain (Fig 1B). In addition, only three amino acids (aa 706–708) with visible density were detected in the Nuc domain, which is composed of 36 amino acids (Fig 1C).

### Overall structure of Cas12g-sgRNA binary complex

**Fig 2:**
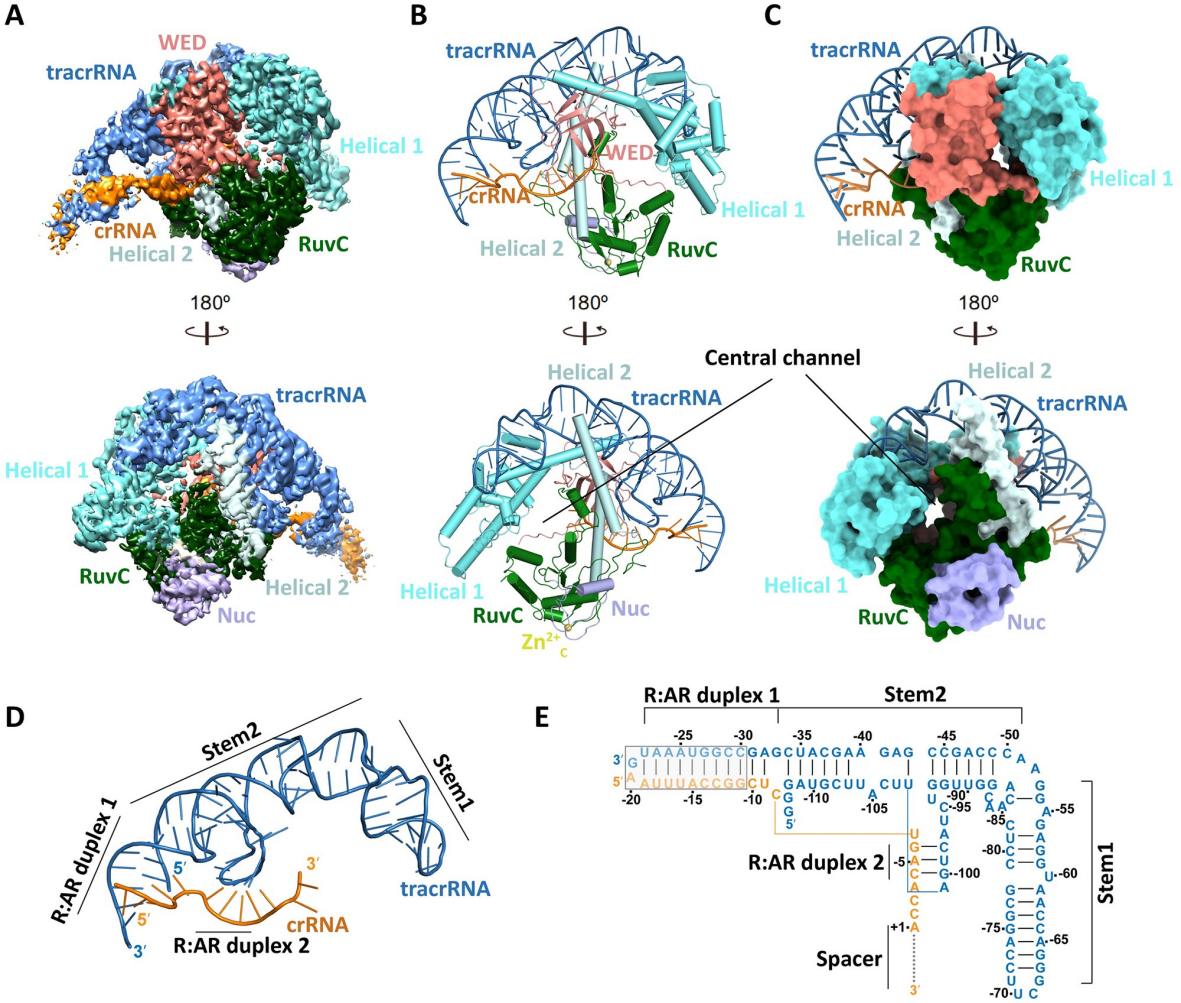
Structure of the Cas12g-sgRNA binary complex. **(A)** Cryo-EM map of the Cas12g binary complex shown in one view and rotated through 180°, with each domain of Cas12g color-coded as in Fig 1A. **(B)** Atomic model of the Cas12g binary complex displayed in two views. **(C)** Surface representations of the Cas12g binary complex shown in the same views as in **(B)**. **(D)** Structure of sgRNA in the Cas12g binary complex. **(E)** Schematic representation of sgRNA, with the disordered region enclosed in the gray box. The ellipsis indicates the invisible region of crRNA.

The Cas12g-sgRNA binary complex was formed by incubating the catalytically inactive Cas12g (D513A) mutant with a 139-nucleotide (nt) sgRNA, which was determined by cryo-EM at a resolution of 3.1 Å and showed a clear density map (Fig 2A). Compared to apo-Cas12g, the REC and NUC lobes in the Cas12g-sgRNA binary complex undergo significant rearrangements, creating a central channel accommodating the sgRNA guide region (Fig 2B and 2C). Additionally, the short Nuc domain, consisting of 36 aa and containing a CCCC-type zinc finger, is visible in the binary complex and was found to be inserted between the RuvC-II and RuvC-III subdomains (S3B and S3E Fig).

The sgRNA used in the complex was generated by fusing a 45-nt crRNA with a 94-nt tracrRNA (Fig 2D). The crRNA comprises a 21-nt repeat region and a 24-nt guide region. The tracrRNA can be divided into two stem regions and two anti-repeat regions, which further form two repeat:anti-repeat (R:AR) duplexes with the crRNA repeat region (Fig 2E). R:AR duplex-1 protrudes away from the core of the complex, lacking direct contact with Cas12g and the nucleotides G(-30)-C(-11) are not modeled due to the weak density. Also, only one nucleotide A (+1) of the crRNA guide region is visible in the present model due to the weak density, suggesting that the crRNA guide region possesses certain flexibility in the binary complex.

### Key interactions between sgRNA and Cas12g

**Fig 3:**
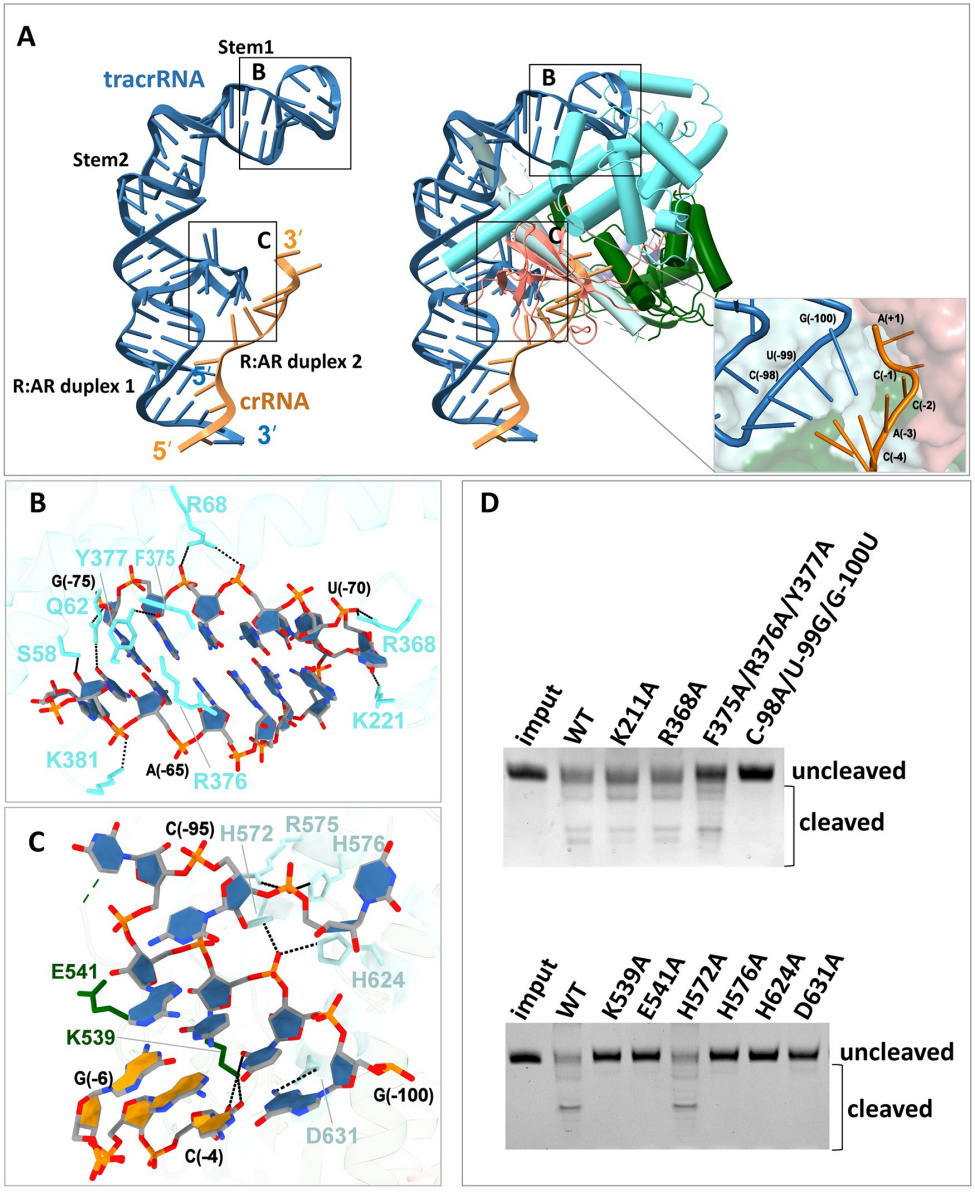
Interaction between Cas12g and sgRNA. **(A)** Ribbon diagram of sgRNA (left panel) and its interaction with the Helical 1, Helical 2 and RuvC domains (right panel). Close-up view of the opening on Cas12g guiding the guide crRNA into the central channel. **(B)** Recognition of the stem1 of sgRNA by the Helical 1 domain of Cas12g. **(C)** Recognition of the R:AR duplex 2 of sgRNA by the Helical 2 and RuvC domain of Cas12g. **(D)** RNA cleavage assay using wild-type Cas12g and Cas12g mutants in combination with sgRNA.

In the Cas12g-sgRNA complex, the sgRNA is bound to the outer concave surface formed by the Helical 1, Helical 2 and WED domains, which establishes extensive charged interactions. The 3’-end nucleotides A (-3)-A (+1) of crRNA are guided from this outer concave surface into the positively charged central channel through an opening between the WED and Helical 2 domains (Fig 3A). The charged interactions are mainly concentrated in stem 1 and the R:AR duplex 2 (Figs 3B, 3C and S8).

Alanine substitutions of key residues 375-FRY-378 decreased the cleavage activity of Cas12g. However, substitutions of residue K221 or R368, which form hydrogen bonds with nucleotides U(-71) and U(-70) of the U-shaped turn of the stem 1 region, had little effect on the cleavage activity of Cas12g. The short R:AR duplex 2 is located at the entrance of the central channel and forms extensive hydrogen bonds with residues from the Helical 2 and RuvC domains (Fig 3D). Mutating R:AR duplex 2-interacting residues K539, E541, H576, D631 and H624 to alanine abolished or severely decreased cleavage activity of Cas12g. However, mutation of H572 did not affect Cas12g cleavage activity. Furthermore, a mismatch of C(-98)A/U(-99)G/G(-100)U in the R:AR duplex 2 abolished the cleavage activity of Cas12g (Fig 3D). Taken together, these findings indicate that both the correct base pairings within R:AR duplex 2 and accurate interactions between Cas12g and R:AR duplex 2 are crucial for Cas12g endonuclease activity.

### Regulation of Cas12g activity by zinc finger motifs

**Fig 4:**
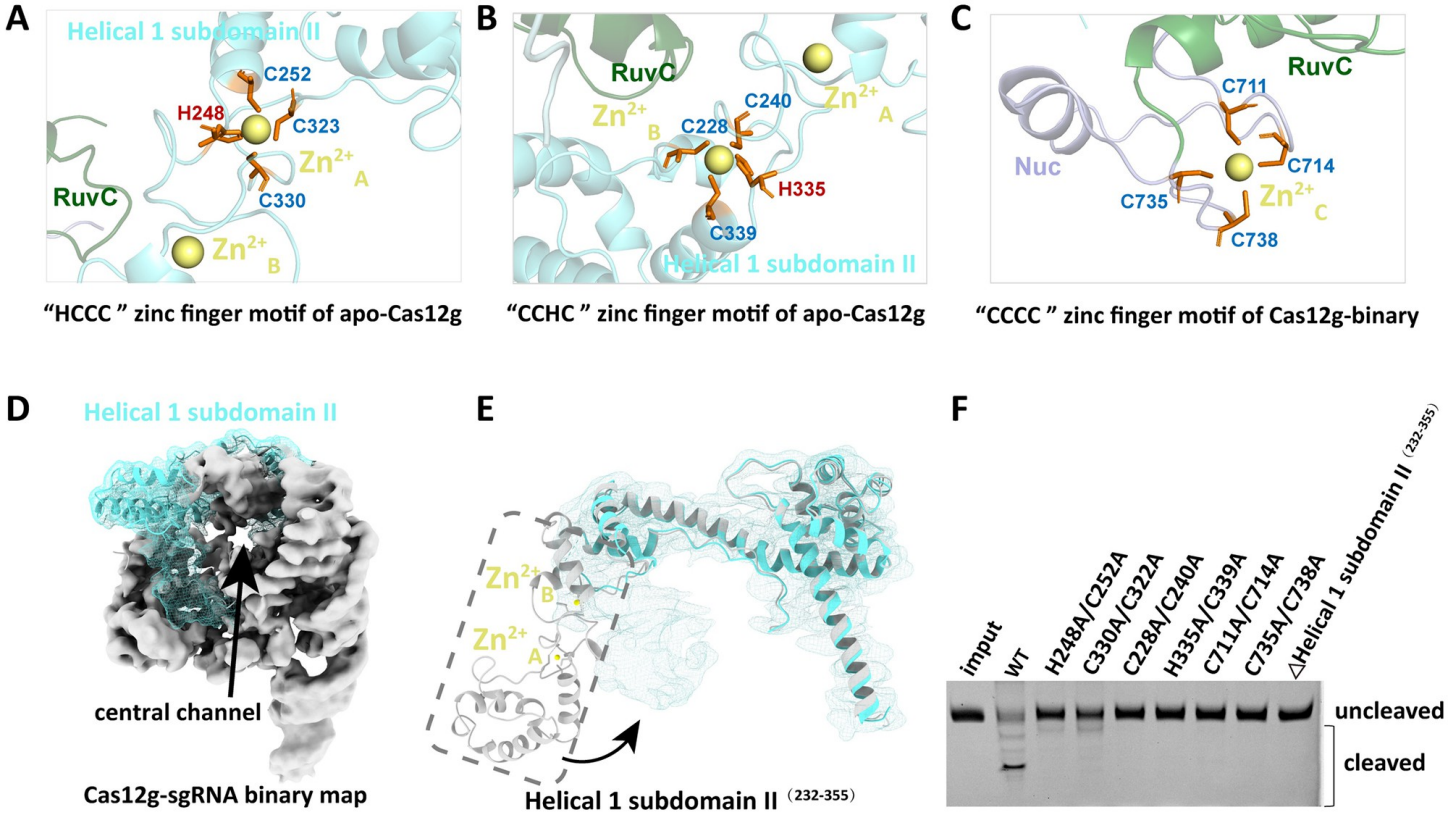
Zinc finger motifs of Cas12g. **(A)** A zinc ion chelated by four residues (H248, C252, C323 and C330) in the Helical 1 subdomain II. **(B)** A zinc ion chelated by four residues (C228, C240, H335 and C339) in the Helical 1 subdomain II. **(C)** A zinc finger motif with four cysteines (C711, C714, C735 and C738) in the Nuc domain. **(D)** Fitting of the unsharpened cryo-EM map with the structure of Cas12g binary complex. Helical 1 domain is colored cyan, and the remaining domains are colored gray. The cryo-EM map color zone with the structure of Cas12g and the density of Helical 1 domain represented by the cyan mesh. **(E)** Superposition of the Helical 1 domain between apo-Cas12g (gray) and Cas12g binary complex (cyan). The EM density of the Helical 1 is shown as cyan mesh. **(F)** RNA substrate cleavage assay using wild-type Cas12g and Cas12g with mutations, as indicated.

In the apo-Cas12g structure, two zinc finger motifs are located at the Helical 1 subdomain II, namely, the HCCC-type zinc finger (His248-Cys252-Cys323-Cys330) and the CCHC-type zinc finger (Cys228-Cys240-His335-Cys339) (Fig 4A and 4B). Each motif coordinates a Zn^2+^ ion and is referred to as Zn^2+^_A_ (HCCC) and Zn^2+^_B_ (CCHC) hereafter, respectively. Interestingly, only two α-helices of the Helical 1 subdomain II are visible, showing discontinuity in the Cas12g-sgRNA binary complex due to the limited resolution. Although the remaining electron density of the Helical 1 subdomain II region (aa 232–355) that contains these two zinc finger motifs cannot be traced in the binary complex, the orientation can be unambiguously discerned using unsharpened density maps (Figs 4D and S2C). Superimposing the Helical 1 domains of apo-Cas12g and binary complex revealed a distinct movement of the subdomain II. Specifically, the region spanning aa 232–355 region of the Helical 1 subdomain II, which is positioned away from the core of apo-Cas12g, swings toward the central channel of the binary complex (Fig 4D and 4E). Deleting the aa 232–355 region was found to abolish the RNase activity of Cas12g, indicating the significant role of this region in substrate cleavage (Fig 4F).

We substituted the adjacent residues on each of these two zinc finger motifs (H248A/C252A, C323A/C330A, C228A/C240A, and H335A/C339A) and tested the target RNA cleavage abilities of these Cas12g mutant variants. As expected, the CCHC zinc finger motif mutations coordinating the Zn^2+^_B_ ion abolish the RNA cleavage activity of Cas12g, similar to the effect observed with the deletion of the aa 232–355 region. Moreover, mutations in the HCCC zinc finger coordinating Zn^2+^_A_ significantly reduced nuclease activity (Fig 4F). Altogether, these results show that these two zinc fingers are crucial motifs involved in target RNA cleavage. Additionally, a third zinc finger motif, the CCCC-type zinc finger (Cys711-Cys714-Cys735-Cys738), is clearly observed in the Nuc domain of the Cas12g binary complex and coordinates the Zn^2+^_C_ ion (Fig 4C). Further, alanine substitutions of these zinc finger residues (C711A/C714A, C735A/C738A) also abolish target RNA cleavage (Fig 4F).

Sequence alignment analysis revealed a high degree of conservation in the zinc finger motifs among the Cas12g family (S4A Fig). To assess the impact of the zinc finger motifs on the thermal stability of Cas12g, a fluorescence thermal shift assay was conducted comparing wild-type Cas12g with its zinc finger mutants. The results demonstrated that the disruption of each of the three zinc finger motifs impacted the thermal stability of the Cas12g protein. Specifically, the zinc finger motif mutants (including H248A/C252A and C323A/C330A) exhibited a noticeable difference (~4.3°C) in melting temperature compared to the wild-type Cas12g (S4B Fig). Overall, these findings suggest that the zinc finger structures not only influence the activity of the Cas12g protein but also play a role in maintaining protein thermal stability.

To further validate the role of zinc finger structures in Cas12g, its nonspecific cleavage activities, including collateral RNase and single-strand DNase, were examined. The analysis revealed that mutations of key residues in the zinc finger motifs, including H248A/C252A, C228A/C240A, H335A/C339A, C711A/C714A and C735A/C738A, completely abolished the collateral RNase and single-strand DNase activities. Additionally, a slight residual cleavage activity was observed in the mutation of C323A/C330A (S4C Fig). These findings are consistent with the observed target RNA cleavage activity and provide further evidence that mutations in the zinc finger motifs can significantly impact the collateral RNase and single-strand DNase activities of Cas12g.

### Conformational rearrangement activating Cas12g endonuclease activity

**Fig 5:**
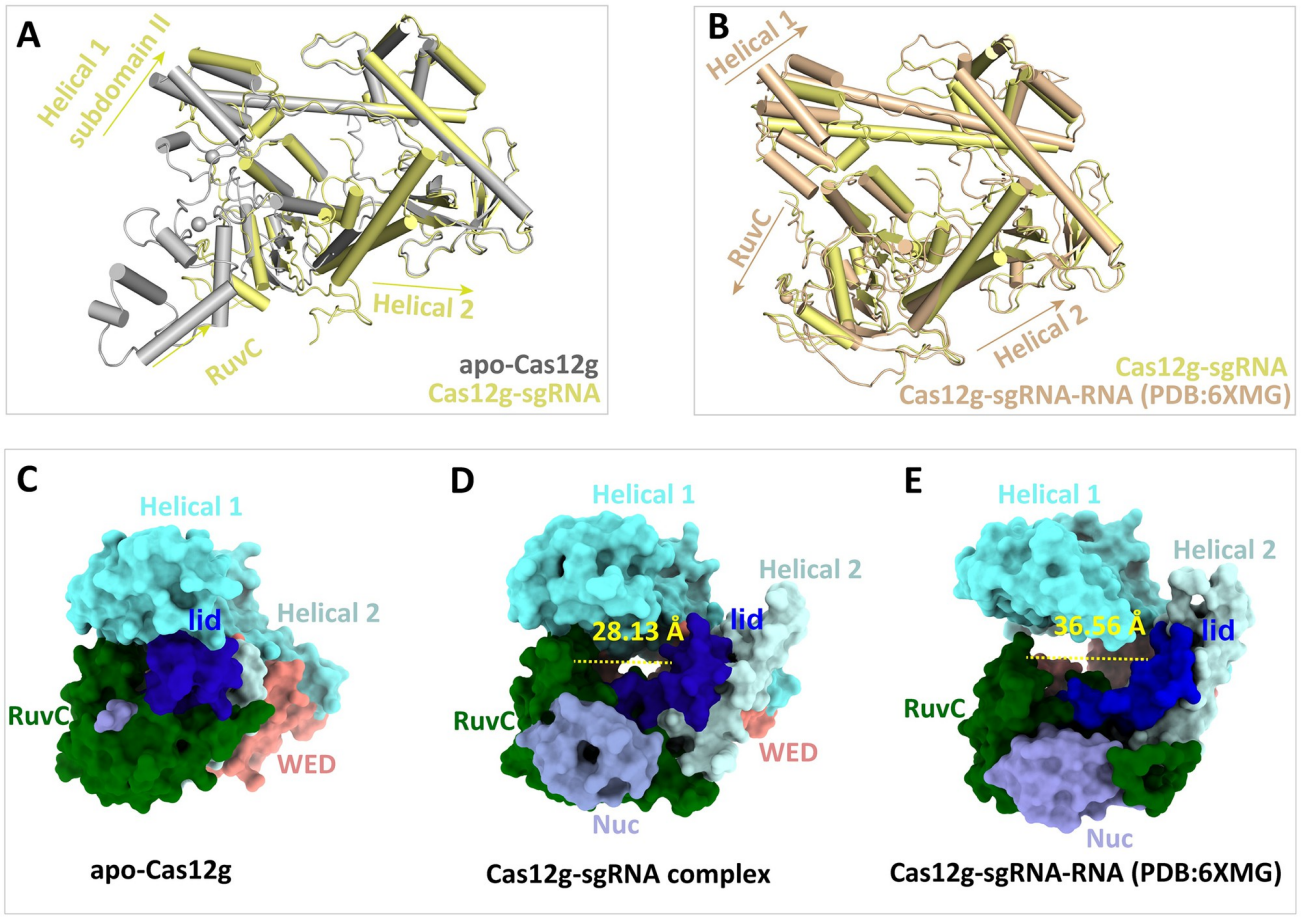
Domain rearrangement of Cas12g. **(A)** Superimposition of apo-Cas12g (gray) and the Cas12g binary complex (pale yellow). **(B)** Superimposition of the Cas12g binary (pale yellow) and ternary (PDB code: 6XMG) (orange) complexes. **(C-E)** Formation of the central channel induced by conformational changes. The aa 655–680 of the RuvC domain constitute the lid motif and are colored in blue. The central channel widens as Cas12g transitions from apo-Cas12g **(C)** to the Cas12g binary complex **(D)** upon sgRNA binding. The central channel further expands with the binding of target RNA **(E)**.

Activation of Cas12g involves significant conformational rearrangements. When comparing apo-Cas12g with the Cas12g binary complex, it is evident that domains within Cas12g undergo a "rigid body" shift upon sgRNA recognition (Fig 5A). This shift is primarily observed in the RuvC, Helical 1 and Helical 2 domains, which move away from the core of Cas12g. A similar phenomenon is also observed during target RNA recognition, as reported in the ternary structure [20] (Fig 5B). These outward movements of the domains lead to the formation of a 23.8 Å-wide central channel from the apo state to the binary complex, providing sufficient space to accommodate the crRNA guide region (Fig 5C and 5D). With the formation of the crRNA-target RNA heteroduplex, the central channel expands to 36.56 Å wide (Fig 5E).

A notable conformational change occurs in the lid motif, which consists of the long helix α23 and the adjacent loop region in the RuvC domain (aa 655–680) (S3G Fig). In the apo state, this motif is located within the central channel. However, during sgRNA recognition, the lid motif undergoes rotation toward the edge of the central channel (Fig 5C and 5D), which facilitates the opening of the exit of the central channel.

**Fig 6:**
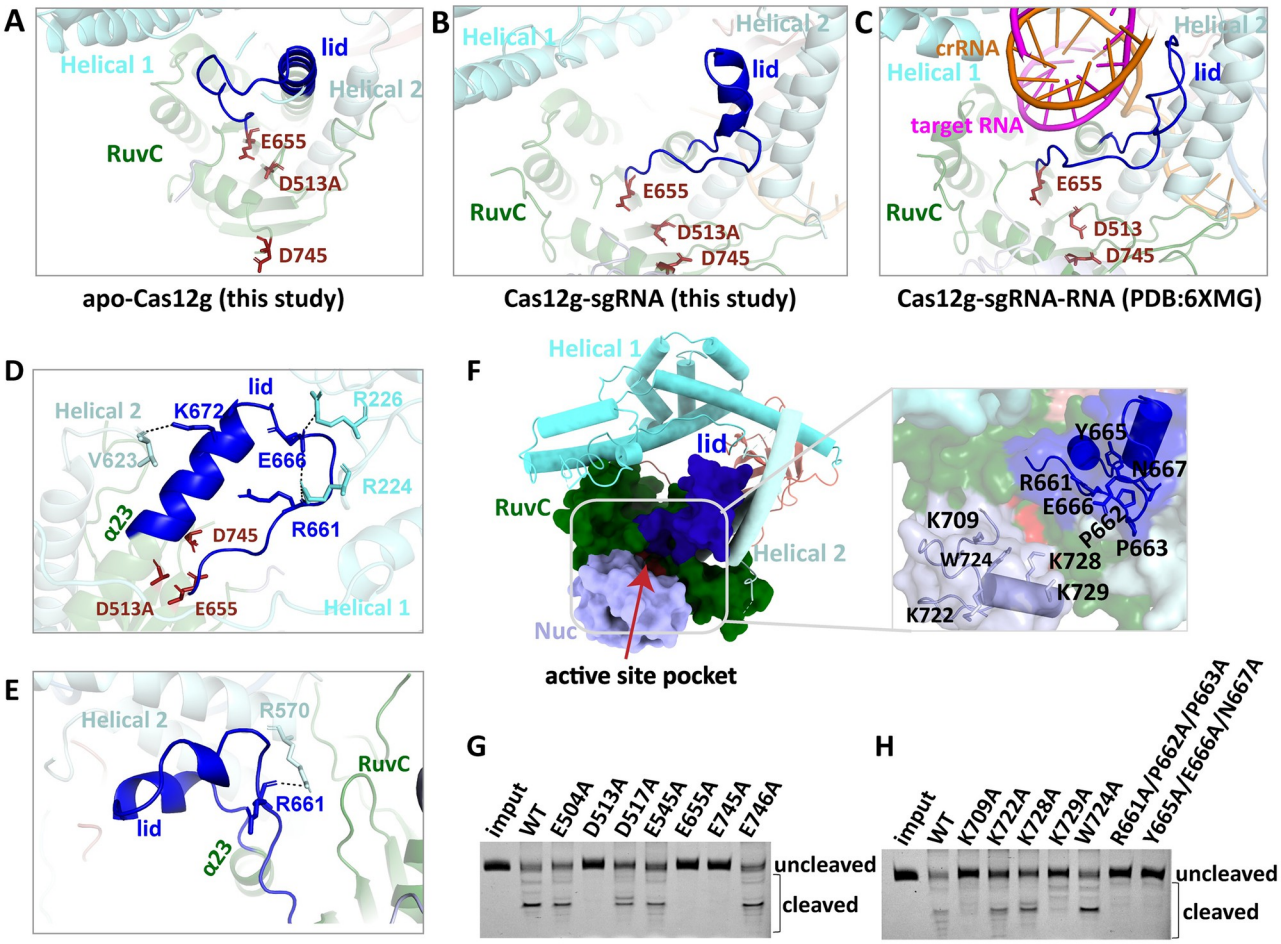
Conformational rearrangement of the lid motif. **(A-C)** The conformations of the lid motif (colored in blue) in apo-Cas12g, the Cas12g binary and ternary (PDB code: 6XMG) complexes. Active residues are colored red. **(D)** Interactions associated with the lid motif in the apo-Cas12g. **(E)** Interactions associated with the lid motif in the Cas12g-sgRNA complex. **(F)** The Nuc domain is attached to the active site, with multiple positively charged residues pointing to the active site. **(G-H)** The substrate RNA cleavage assay using wild-type Cas12g and Cas12g with mutations, as indicated. Alanine substitution of the Asp and Glu residues in the RuvC domain **(G)** and the alanine substitution of the key residues in the lid motif and Nuc domain **(H)**.

Importantly, the conformational change of the lid motif contributes to the formation of the central channel and plays a key role in regulating substrate cleavage. An activated catalysis pocket with the three Asp-Glu-Asp conservative nuclease sites is observed in Cas12 systems. In Cas12g, three conserved residues, namely, Asp513, Glu655 and Asp745, located in the RuvC domain have been identified as the nuclease active site via alanine substitution of Asp and Glu residues in the RuvC domain (Fig 6G). Analysis of different structures representing various stages of the reaction reveals that the lid motif acts as a substrate-catalytic regulator in activating Cas12g (Fig 6).

In the inactive state of apo-Cas12g, the lid motif is positioned above the catalytic active site, obstructing the catalytic core region and preventing substrate binding and cleavage (Fig 6A). Recognition and binding of cognate sgRNA by Cas12g induces rotation of the lid motif by a partial helix-to-loop transition of helix α23, which partially uncovers the RuvC active site and opens the substrate-binding groove (Fig 6B). Subsequently, recognition of the target RNA induces a further helix-to-loop transition of the lid motif and movement to accommodate the guide-target RNA heteroduplex (Fig 6C). Target RNA binding causes further conformational adjustments of the lid motif, fully exposing the RuvC active site and facilitating substrate cleavage.

In apo-Cas12g, the lid motif interacts with the Helical 1 subdomain II and Helical 2 domains and is held in place above the active site through hydrogen bonding between residues R661-R224, E666-R226 and K672-V632 (Fig 6D). This conformation maintains Cas12g in an inactive state, preventing the catalytic active site from accessing and cleaving substrates. However, when Cas12g binds with sgRNA, the guide crRNA enters the central channel, which rearranges the Helical 1 subdomain II and Helical 2 domains and dissociates the hydrogen bonds between R661-R224, E666-R226 and K672-V632. As a result, only an interaction between R661-R570 is observed in the state with sgRNA binding, leaving the catalytic pocket open (Fig 6E and 6F). In addition, alanine substitutions of key residues from the lid motif (R661A/P662A/P663A, Y665A/E666A/N667A) and Nuc domain (K709A, K722A, K728A, and K729A) significantly impair the nuclease activity of Cas12g (Fig 6H), indicating that the lid motif and Nuc domain may be involved in binding and stabilization of the guide-target RNA heteroduplex and facilitating the entry of target RNA into the RuvC active site.

## Discussion

Cas12g is a newly discovered RNA-guided RNase identified through the analysis of the prokaryotic metagenome database [12, 25]. Unlike other type V Cas effectors that primarily recognize DNA substrates, such as Cas12a [26], Cas12b [22], Cas12e [27] and Cas12i [16,17], Cas12g specifically recognizes RNA substrates [12]. The structures of apo-Cas12g and the Cas12g binary complex presented in this study shed light on the rearrangement of multiple domains within Cas12g upon sgRNA binding and their roles in preparing for target RNA cleavage.

Although a previous study suggested that the C-terminal region of Helical 1 ^(232–355)^ plays an essential regulatory role in the activation of Cas12g, the structural characteristics remain unclear due to limited resolution [24]. Fortunately, the structure of apo-Cas12g determined in this study provides an intact view of the Helical 1 subdomain II, which consists of helices α9–α17. Within this subdomain, the Cys3His (CCHC)-type and HisCys3 (HCCC)-type zinc fingers, each coordinating a Zn^2+^, were identified. Structural analyses of apo-Cas12g, its binary complex and the previously reported ternary complexes revealed that the Helical 1 subdomain II undergoes distinct movement and that the Helical 1 subdomain II region (aa 232–355), which is located away from the protein core in apo-Cas12g, can move toward the exit of the central channel in the Cas12g binary complex, eventually attaching to the guide-target RNA heteroduplex in the Cas12g ternary complex [20] (S6 Fig). The activation process of Cas12g is accompanied by conformational rearrangement of the Helical 1 subdomain II. The two zinc finger motifs within this subdomain are likely involved in recognizing the guide-target RNA heteroduplex. Another zinc finger motif is located within the Nuc domain, adjacent to the RuvC domain. Sequence alignment revealed that these three zinc finger motifs exhibit a high degree of conservation within the Cas12g family (S4A Fig). Cleavage assays confirmed that disrupting any of these three zinc finger motifs abolishes or decreases the enzymatic activity of Cas12g, including collateral RNase and single-strand DNase activities. Therefore, these three zinc finger motifs play a crucial role in the cleavage activity of the Cas12g effector.

Zinc finger motifs exhibit notable diversity, and some have been shown to interact with various molecules other than DNA, including RNA, proteins and lipids [28]. Classical zinc fingers, such as Cys2His2 (CCHH), Cys3His (CCHC) and Cys4 (CCCC), are characterized by two β-strands and one α-helix, while nonclassical zinc fingers have a more random structure, with a high degree of coiled loops [29]. Nevertheless, the classical types of CCCC and CCHC and the nonclassical type of HCCC zinc finger motifs in Cas12g reported in this study display many loop structures and lack β-strands. These three types of zinc finger motifs have been previously shown to not only recognize DNA strands [30–33] but also interact with double-stranded RNA (dsRNA) and single-stranded RNA (ssRNA) in different modes [30,34–37]. In addition, the CCHC and CCCC zinc finger motifs possess a high binding affinity for RNA, especially ssRNA [36,38–40]. Zinc finger-mediated recognition of RNA usually involves backbone recognition of dsRNA and recognition of customized RNA base recognition [37,39]. Further investigations are required to elucidate how the zinc finger motifs in the Helical 1 and the Nuc domains of Cas12g recognize the guide-target RNA heteroduplex. In general, zinc finger-dependent target RNA cleavage by CRISPR–Cas12g presents new possibilities for RNA detection, editing and/or engineering. Our study provides a mechanistic model for the RNase activities of Cas12g, outlining the process of Cas12g activation and RNA substrate cleavage. However, unanswered questions remain. Thus, future studies should aim to unravel the precise mechanisms via which the zinc finger motifs of Cas12g contribute to the recognition of target RNA and guide-target RNA heteroduplexes.

Previous studies on type V Cas effectors, including Cas12a [21], Cas12b [22], Cas12i [16] and Cas12j [23], have shown that target binding triggers a conformational rearrangement of the lid motif, fully exposes the RuvC active sites of Cas12 effectors and activates them. Notably, in this present study, the structural analysis of apo-Cas12g and the binary complex reported revealed that the lid motif undergoes rotation and a helix-to-loop transition upon binding of sgRNA, partially exposing the RuvC active site. Importantly, this conformational rearrangement of the lid motif occurs before the binding of target RNA. Based on these observations, we propose a mechanistic model to illustrate the process of Cas12g activation and RNA substrate cleavage (S7 Fig).

Although Cas12g exhibits RNA-guided RNase activity, its architecture and functional domains differ from those of Cas effectors of type VI systems (i.e., Cas13a, Cas13b, and Cas13d) [41–43], which specifically cleave RNA substrates using two HEPN (i.e., in higher eukaryotes and prokaryotes nucleotide-binding) domains. Functionally, all Cas13 effectors are crRNA-guided RNases with two distinct and independent catalytic centers that can process pre-crRNA into mature crRNA and cleave target RNA via the two typical R-X_4_-H motifs in HEPN domains [44,45]. In contrast, Cas12g appears to be unable to process its pre-crRNA, suggesting that additional endogenous factors might be required for crRNA maturation [12]. In particular, Cas12g contains a single RuvC domain that can cleave the ssRNA target and collateral ssDNA or ssRNA. The RuvC domain, which is highly conserved in Cas12 effectors, is primarily involved in cleaving DNA substrates using three acidic residues (Asp-Glu-Asp) as nuclease sites in the presence of divalent metal ions. In contrast, type VI CRISPR–Cas systems, particularly Cas13 systems (except Cas13d and Cas13X), require a protospacer flanking sequence (PFS) in the target ssRNA, similar to the role of PAM in other systems for RNA cleavage. However, the Cas12g effector does not require a PFS for substrate interference [12]. Due to the lack of PAM or PFS restrictions and the ease of vector packaging and intracellular delivery, Cas12g shows promise as an RNA-targeting tool.

## Materials and methods

### Cas12g expression and purification

The codon-optimized sequence of the Cas12g full-length gene (S1 Table) was cloned between BamHI and XhoI sites of the pET-30b vector with a C-terminal 6×His-tag and transformed into *E*. *coli* Rosetta (DE3) competent cells. The bacterial cultures were grown in LB medium at 37°C until OD_600_ reached ~0.8–1.0, after which the overexpression of the recombinant protein was induced with 0.2 mM IPTG at 16°C for 12 h. Afterward, the cells were pelleted by centrifugation and resuspended in ice-cold lysis buffer (50 mM Tris-HCl (pH 8.5), 1 M NaCl, 5 mM β-ME, 1 mM PMSF). The bacteria were then sonicated and centrifugated to separate the supernatant from cell debris. The clarified supernatant was loaded onto a Ni-NTA affinity column and allowed to flow through the column at a flow rate of 0.8 mL/min. After extensive washing, the target protein was eluted with elution buffer (50 mM Tris-HCl (pH 8.5), 150 mM NaCl, 200 mM imidazole, 1 mM PMSF). The target protein was then purified with a HiTrap Heparin column (GE Healthcare) using a salt gradient between buffer A (50 mM Tris-HCl (pH 8.5), 500 mM NaCl, 1 mM MgCl_2_, 1 mM DTT) and buffer B (50 mM Tris-HCl (pH 8.5), 1.5 M NaCl, 1 mM MgCl_2_, 1 mM DTT), followed by pooling and concentrating the fractions containing target protein in buffer C (50 mM Tris-HCl (pH 8.5), 150 mM NaCl, 1 mM MgCl_2_, 1 mM DTT). The concentrated target protein was further purified with Mono S 10/100 column (GE Healthcare) using salt gradient between buffer D (50 mM Tris-HCl (pH 8.5), 150 mM NaCl, 1 mM MgCl_2_, 1 mM DTT) and buffer E (50 mM Tris-HCl (pH 8.5), 1.5 M NaCl, 1 mM MgCl_2_, 1 mM DTT). Fractions containing purified target protein were pooled, concentrated, and purified by size-exclusion chromatography (Superdex 200 Increase 10/300 GL column, GE Healthcare) in buffer D (50 mM Tris-HCl (pH 8.5), 150 mM NaCl, 1 mM MgCl_2_, 1 mM DTT). Peak fractions were concentrated and stored at -80°C until further use.

Selenomethionine (SeMet)-labeled Cas12g (D513A) mutant was expressed in *E*. *coli* Rosetta (DE3) cells cultured in M9 minimal medium supplemented with SeMet, Lys, Phe, Thr, Val, Leu, and Ile. The SeMet-labeled Cas12g D513A mutant was purified following the procedure described above.

### *In vitro* transcription and purification of RNA

The DNA fragments serving as templates for sgRNA and target RNA sequences (S2 Table) were synthesized and cloned separately into modified pUC-119 vectors between EcoRI and HindIII sites. The recombinant plasmids were amplified in *E*. *coli* DH5α cells (Novagen). Amplified plasmids were extracted, linearized by HindIII, and then purified using phenol-chloroform extraction and ethanol precipitation. The linearized DNA templates were used to carry out in vitro RNA transcription with homemade T7 RNA polymerase. Transcribed RNA products were resolved in denaturing polyacrylamide gel containing 8 M urea. Desired RNA bands were excised from the gel and eluted using the Elutrap system (GE Healthcare). The eluted RNA was desalted, concentrated by ethanol precipitation, and dissolved in diethylpyrocarbonate (DEPC)-treated H_2_O. All RNA samples were stored at -80°C.

### *In vitro* assembly of the Cas12g-sgRNA binary complex

Before assembling the binary complex, sgRNA samples stored at -80°C were thawed at 4°C, denatured at 75°C for 5 min, and then allowed to be cooled at 4°C. Afterward, Cas12g and sgRNA were mixed in buffer D (50 mM Tris-HCl (pH 8.5), 150 mM NaCl, 1 mM MgCl_2_, 1 mM DTT) at a molar ratio of 1:1.2 and incubated at 16°C for 1 hour. The mixture was first purified with the Q column to remove excess sgRNA, followed by further purification using the Superdex 200 Increase 10/300 GL column. Fractions containing purified and homogenous Cas12g-sgRNA binary complex were pooled and concentrated.

### Circular dichroism spectroscopy

Purified wild-type and mutant Cas12g proteins were detected using Chirascan (Applied Photophysics) at room temperature, with a wavelength range of 190–240 nm in the far-ultraviolet region. The concentration of the samples was diluted to 0.2 mg/ml. Quartz cuvettes with a path length of 0.1 cm were used for the measurements. The baseline was established using the buffer spectrum, and the spectra were obtained by subtracting the baseline. Each sample was measured three times for repeatability.

### Fluorescence thermal shift assay

Melting curves of effector proteins were determined through differential scanning fluorimetry (DSF). It was determined by monitoring the changes in tryptophan fluorescence intensity or the tryptophan fluorescence ratio (i.e., the 350/330 nm ratio) in the target protein. The wild-type Cas12g and its zinc-finger mutants proteins are diluted to a concentration of 1 mg/ml respectively. Aspirate the prepared sample without introducing any bubbles using a pipette and transfer it to the sample holder. Perform fluorescence monitoring at a rate of 1°C/min, within a temperature range of 20°C to 95°C. The first derivative of the raw fluorescence data was taken in order to determine the Tm of the effector. Each sample was measured three times for repeatability.

### Sequence alignment

Download the amino acid sequences of Cas12g from the NCBI database and perform multiple sequence alignment using the Clustal Omega software (http://www.ebi.ac.uk/Tools/msa/clustalo/).

### Crystallization, X-ray data collection, and structure determination

Crystals of SeMet-labeled apo-Cas12g were screened and optimized with commercial and homemade sparse-matrix crystallization screening kits using the hanging drop vapor diffusion method at 16°C. A Mosquito crystallization robot (SPT Labtech) performed primary screening of crystallization conditions. Diffraction-quality crystals were grown in 0.1 M BIS-TRIS (pH 6.5) and 25% polyethylene glycol 3,350 (w/v) at the protein concentration of A280 nm ≈ 10. Harvested crystals were cryoprotected in 20% (v/v) glycerol and flash-frozen in liquid nitrogen.

X-ray diffraction data were collected using a CCD detector at the beamline BL-17U1 of the Shanghai Synchrotron Radiation Facility (SSRF). Diffraction datasets were processed using XIA2 [46], followed by CCP4 [47]. The crystal structure of SeMet-labeled apo-Cas12g was solved by the single-wavelength anomalous diffraction method using the PHENIX software [48]. Model building was carried out in COOT [49], and model refinement was carried out using REFMAC in CCP4 format. Data collection and refinement statistics are listed in S3 Table.

### Cryo-EM sample preparation and data collection

5 μL of the complex at 3 mg/ml concentration was loaded onto a freshly glow discharged (45 s at 15 mA) holey carbon grid (Quantifoil Au R1.2/1.3) before sample application. Then, the excess liquid is removed by Whatman #1 filter paper at a blot force of 4 s at a blot force of -3 at 4°C, and 100% humidity using a Vitrobot Mark IV plunger (ThermoFisher Scientific) and plunge-frozen in liquid ethane. The prepared frozen-hydrated specimen was loaded on an FEI Titan Krios transmission electron microscope equipped with a Gatan K2 Summit direct detector and Gatan BioQuantum energy filter used for the Date collection. 5,730 movies were collected using SerialEM software that operated the 300 kV transmission electron microscope at a nominal magnification of 165,000× with a pixel size of 0.84 Å, and the movies were recorded with a defocus range of -1.2 to -1.8 μm in super-resolution mode.

### Cryo-EM data processing

The 32 frames of each movie in a super-resolution model were aligned, dose-weighted, and summed using MotionCor2 [50], resulting in summed micrographs in a pixel size of 0.84 Å per pixel. Expect the MotionCorrection of raw movies, and the other date processing was completed in Relion/3.1.2 [51]. Contrast transfer function (CTF) parameters of the micrographs with dose-weighting were estimated using CTFFIND-4.1 [52] and applied to further processing. About 2,700 particles from the manual picking underwent one round of reference-free 2D classification that generated template images for reference-2D classification of all micrographs. After three rounds of reference-2D classification, 570,000 particles were selected from well-defined particle images and used to generate the 3D initial model. After that, 3D classification was performed without imposing symmetry by using the initial reference model. 570,000 particles were classified into 5 classes, and the best class with intact Cas12g-sgRNA was further selected for the 3D classification. The 3D classification shows that the complex adopts a single conformation. The remaining 88,696 particles were selected for a 3D auto-refinement converging at 3.1 Å resolution. The reconstruction resolutions were determined based on the gold-standard Fourier shell correlation (FSC) 0.143 criterion with the high-resolution noise substitution. Focused 3D refinement, focused 3D classification, and 3D refinement was performed sequentially around the Helical 1 subdomain II ^(232–355)^ region to improve the local map quality.

### Model building, refinement, and validation

Structural models of individual domains in apo-Cas12g were docked into the EM density of Cas12g-sgRNA binary complex in UCSF Chimera [53], and then refined in COOT [49] and PHENIX [48] based on the EM map in real space with secondary structure and geometric constraints. The structural model was verified by comprehensive validation included in the PHENIX package. S4 Table summarizes the model statistics.

### *In vitro* target RNA cleavage assays

First, the binary complex was assembled by mixing purified Cas12g with sgRNA at a molar ratio of 4:1 in assay buffer (25 mM Tris-HCl (pH 8.5), 150 mM NaCl 150mM, 10 mM MgCl_2_), and the sample was incubated at 37°C for 30 min. Afterward, target RNA was added to the prepared binary complex using the same molar ratio as sgRNA, and the cleavage reaction was carried out at 37°C for 30 min. Reactions were terminated by adding EDTA (final concentration of 100 mM) and Proteinase K (final concentration of 0.8 mg/ml) at 37°C for 30 min. Reaction samples were run on 20% PAGE TBE-urea denaturing gels stained with GelRed Nucleic Acid Gel Stain and the results were visualized using Alpha Innotech (Fluouchem TM). Cleavage assays were conducted in triplicate.

## Supporting information

S1 Fig 3D reconstruction of the Cas12g-sgRNA binary complex. **(A)** Representative electron micrograph of Cas12g-sgRNA complex embedded in vitreous ice on a Quantifoil grid. The image shows a homogeneous distribution of protein. **(B)** Representative views of 2D class averages. The 2D averages shows clearly elements of secondary structure, revealing the high resolution of the data. **(C)** Flowchart of Cryo-EM data processing and 3D reconstruction of the Cas12g-sgRNA binary complex. **(D)** FSC curves at 0.143 of the final reconstruction of Cas12g-sgRNA binary complex. **(E)** Local resolution map calculated using Relion for Cas12g-sgRNA binary complex.
(TIF)

S2 Fig Detailed cryo-EM density map of the Cas12g-sgRNA complex with final atomic model fitted in. **(A)** Cryo-EM map and model of the Cas12g-sgRNA complex with each domain of Cas12g color coded as in Fig 1**(A)**. **(B)** Fitting of nucleic acids to the corresponding cryo-EM map. The atomic models are shown in stick with crRNA and tracrRNA. The crRNA strand and tracrRNA colored in orange and sky blue, respectively. **(C)** Fitting of the Helical 1 domain. Despite the unresolvable structure Helical 1 subdomain II (aa 232–355) region, its position in the Cas12g-sgRNA complex can be determined based on the density. **(D-G)** Fitting of the Helical 2 **(D)**, WED **(E)**, RuvC **(F)** and, Nuc domain **(G)**.
(TIF)

S3 Fig Structures of individual domains of Cas12g. **(A)** Overall structure of apo-Cas12g. **(B)** Overall structure of the Cas12g binary complex. **(C)** The structure of Helical 1 domain (right). The SAD map around Helical 1 subdomain II discussed in article shown in left. **(D-G)** Structures of Helical 2, Nuc, WED and RuvC domains of Cas12g. Domains are colored according to Fig 1**(A)**.
(TIF)

S4 Fig The lid motif of Cas12g. **(A)** The alignment of the amino acid sequences of Cas12g from different bacterial strains. The amino acids related to the zinc finger motifs are marked with black boxes. **(B)** Circular dichroism (CD) spectra of wild-type and mutants of zinc finger motifs in Cas12g. **(C)** Collateral cleavage of unrelated ssDNA (left) and ssRNA (right) by Cas12g. The results shown are representative of three experiments.
(TIF)

S5 Fig Circular dichroism (CD) spectra of wild-type and mutants of zinc finger motifs in Cas12g. (TIF)

S6 Fig The location of Helical 1 subdomain II (aa 232–355) region in Cas12g-sgRNA-target RNA complex. **(A)** The cryo-EM map of the Cas12g-sgRNA-target RNA complex (PDB code: 6XMG). Helical 1 subdomain II (aa 232–355) region is marked with a red box. **(B)** Structural representation of Helical 1 subdomain II (aa 232–355) region in Cas12g-sgRNA-target RNA complex (PDB code: 6XMG).
(TIF)

S7 Fig Schematic model of the mechanism for conformational activation of the Cas12g RNase activity. Each domain of Cas12g color coded as in Fig 1**(A)**.
(TIF)

S8 Fig Schematic of sgRNA recognition by Cas12g. Domains and residues are colored according to Fig 1**(A)**.
(TIF)

S9 Fig SDS-PAGE analysis of the wild-type and mutants of Cas12g protein. (TIF)

S1 Table DNA coding sequence for Cas12g used in this study. (DOCX)

S2 Table RNA transcription template used in this study. (DOCX)

S3 Table X-ray crystallography data collection and refinement statistics. (DOCX)

S4 Table Statistics of cryo-EM data of the Cas12g binary complex and structure refinement. (DOCX)
