# Supplementary figures and images for: Structural transitions upon guide RNA binding and their importance in Cas12g-mediated RNA cleavage
Source: PLoS Genet. 2023 Sep 20;19(9):e1010930. doi: 10.1371/journal.pgen.1010930 (PMC10511118; doi:10.1371/journal.pgen.1010930)

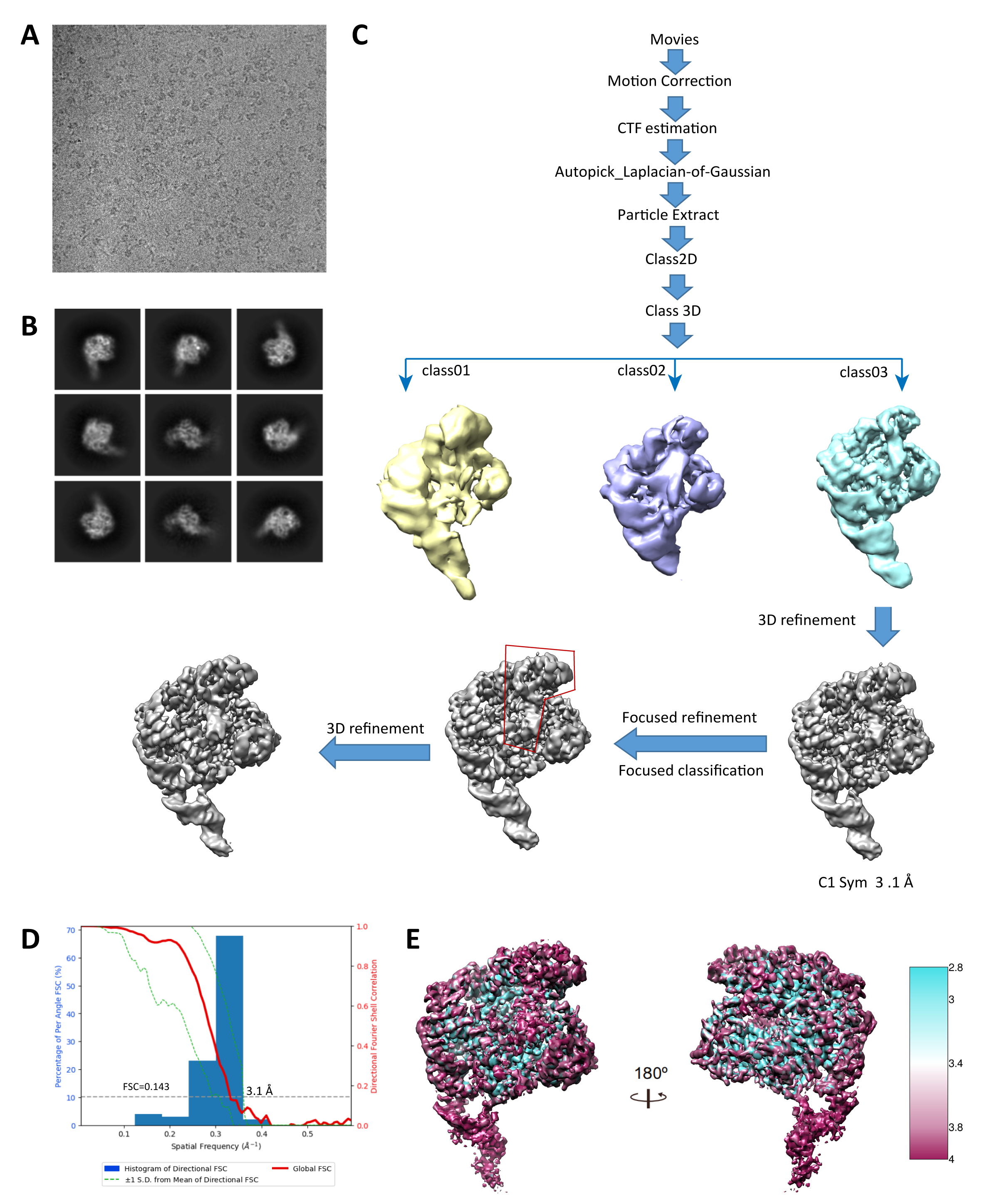

Supplement: S1 Fig — (A) Representative electron micrograph of Cas12g-sgRNA complex embedded in vitreous ice on a Quantifoil grid. The image shows a homogeneous distribution of protein. (B) Representative views of 2D class averages. The 2D averages shows clearly elements of secondary structure, revealing the high resolution of the data. (C) Flowchart of Cryo-EM data processing and 3D reconstruction of the Cas12g-sgRNA binary complex. (D) FSC curves at 0.143 of the final reconstruction of Cas12g-sgRNA binary complex. (E) Local resolution map calculated using Relion for Cas12g-sgRNA binary complex. (TIF) [file pgen.1010930.s001.tif]

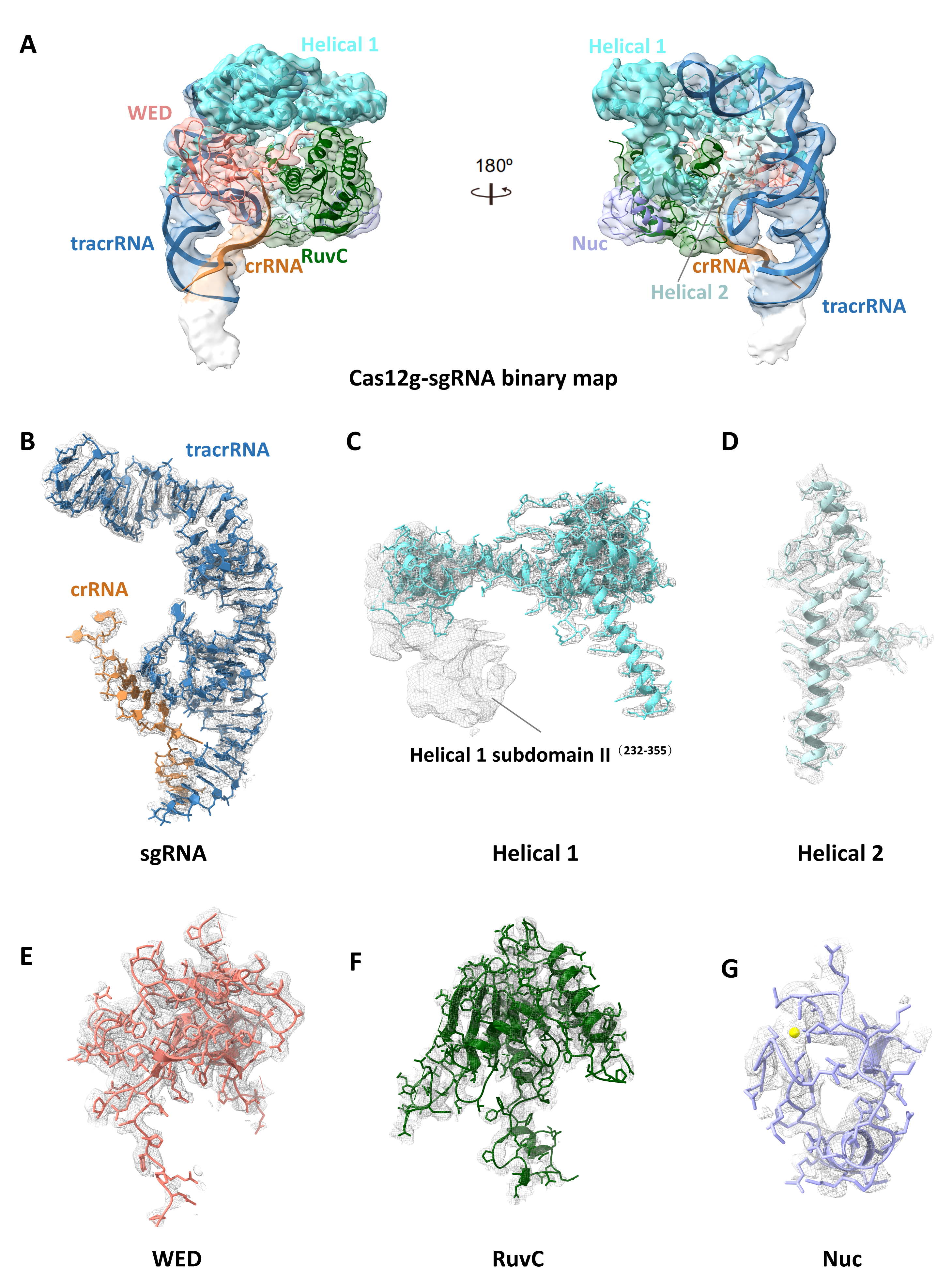

Supplement: S2 Fig — (A) Cryo-EM map and model of the Cas12g-sgRNA complex with each domain of Cas12g color coded as in Fig 1(A). (B) Fitting of nucleic acids to the corresponding cryo-EM map. The atomic models are shown in stick with crRNA and tracrRNA. The crRNA strand and tracrRNA colored in orange and sky blue, respectively. (C) Fitting of the Helical 1 domain. Despite the unresolvable structure Helical 1 subdomain II (aa 232–355) region, its position in the Cas12g-sgRNA complex can be determined based on the density. (D-G) Fitting of the Helical 2 (D), WED (E), RuvC (F) and, Nuc domain (G). (TIF) [file pgen.1010930.s002.tif]

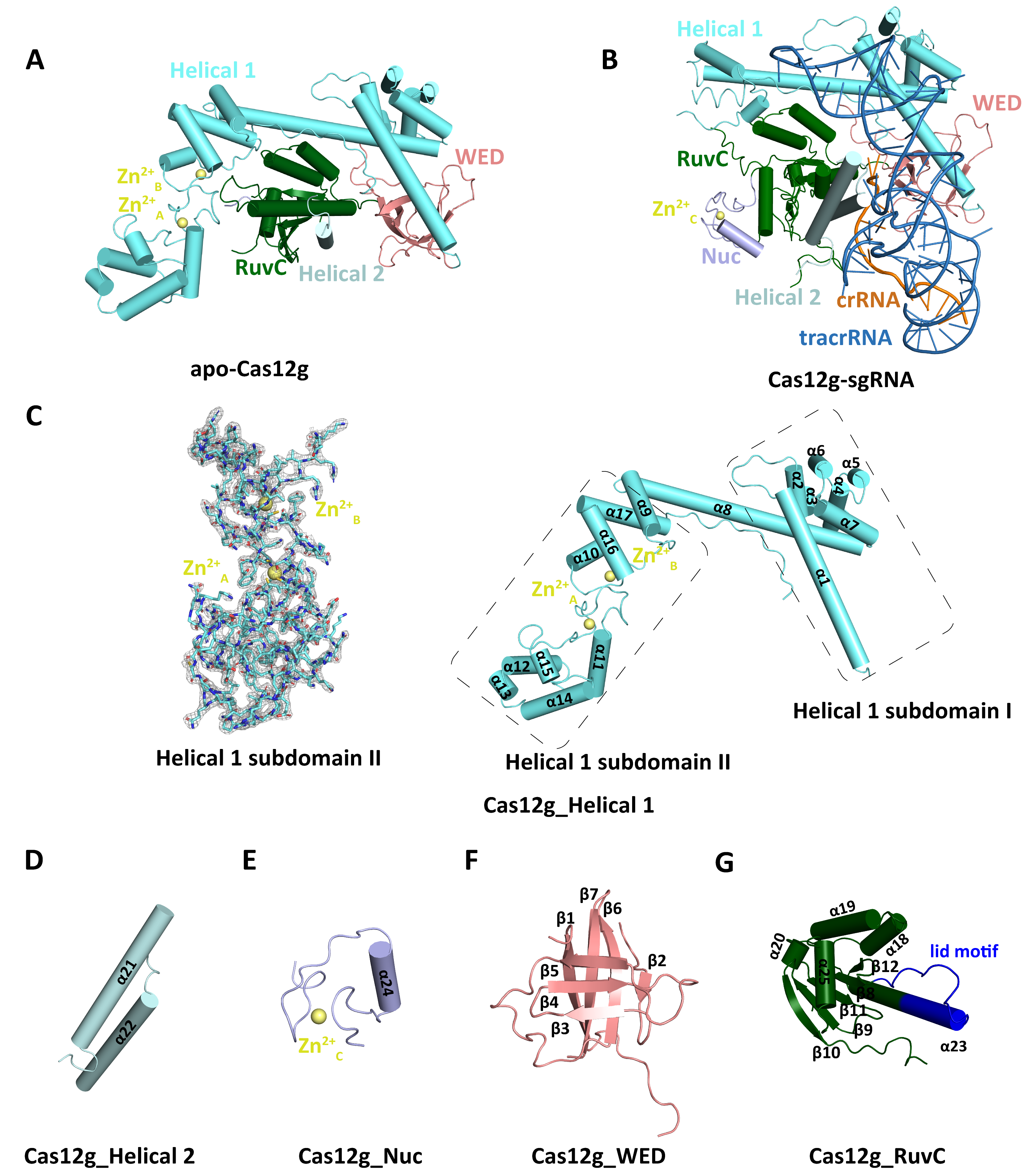

Supplement: S3 Fig — (A) Overall structure of apo-Cas12g. (B) Overall structure of the Cas12g binary complex. (C) The structure of Helical 1 domain (right). The SAD map around Helical 1 subdomain II discussed in article shown in left. (D-G) Structures of Helical 2, Nuc, WED and RuvC domains of Cas12g. Domains are colored according to Fig 1(A). (TIF) [file pgen.1010930.s003.tif]

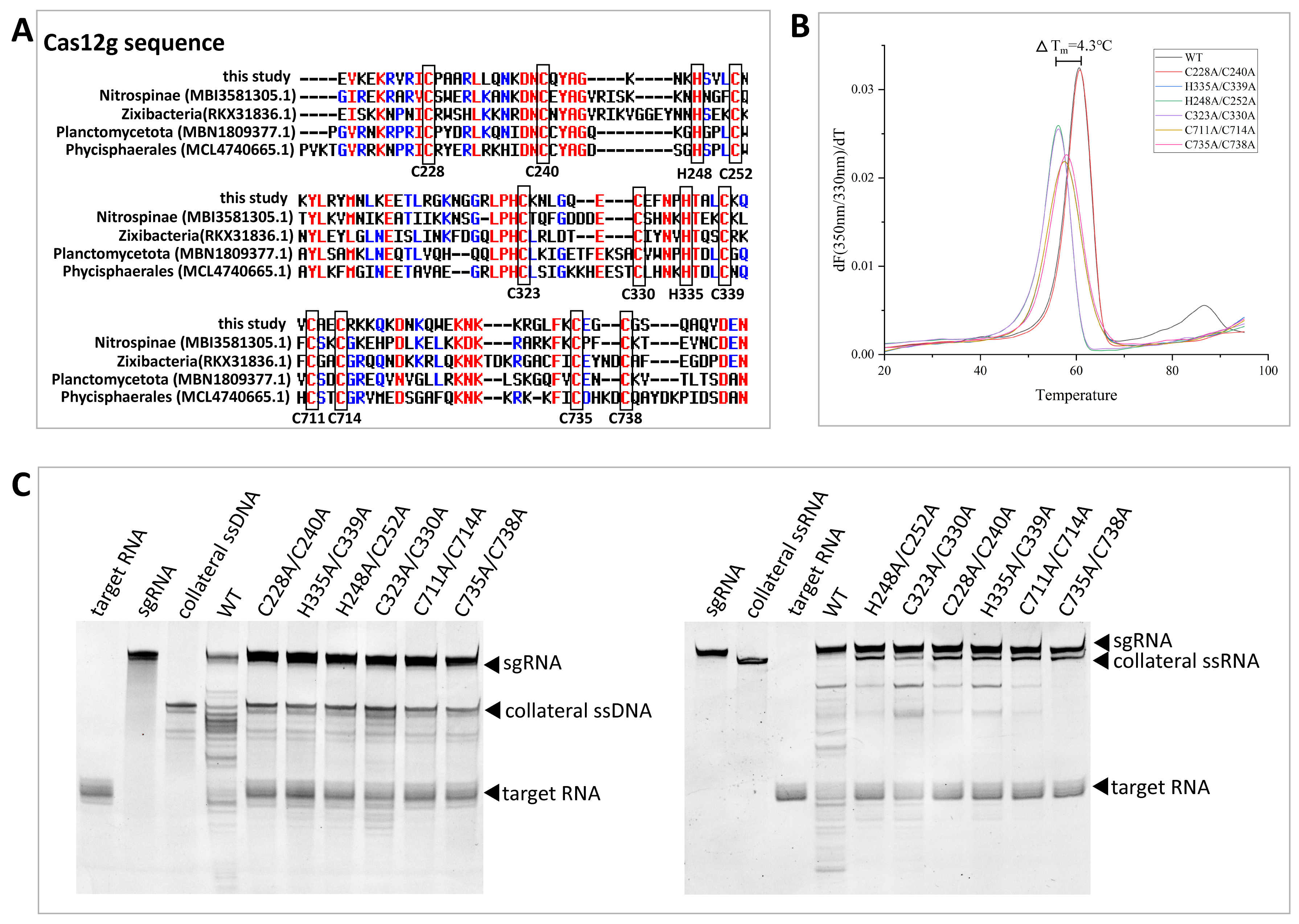

Supplement: S4 Fig — (A) The alignment of the amino acid sequences of Cas12g from different bacterial strains. The amino acids related to the zinc finger motifs are marked with black boxes. (B) Circular dichroism (CD) spectra of wild-type and mutants of zinc finger motifs in Cas12g. (C) Collateral cleavage of unrelated ssDNA (left) and ssRNA (right) by Cas12g. The results shown are representative of three experiments. (TIF) [file pgen.1010930.s004.tif]

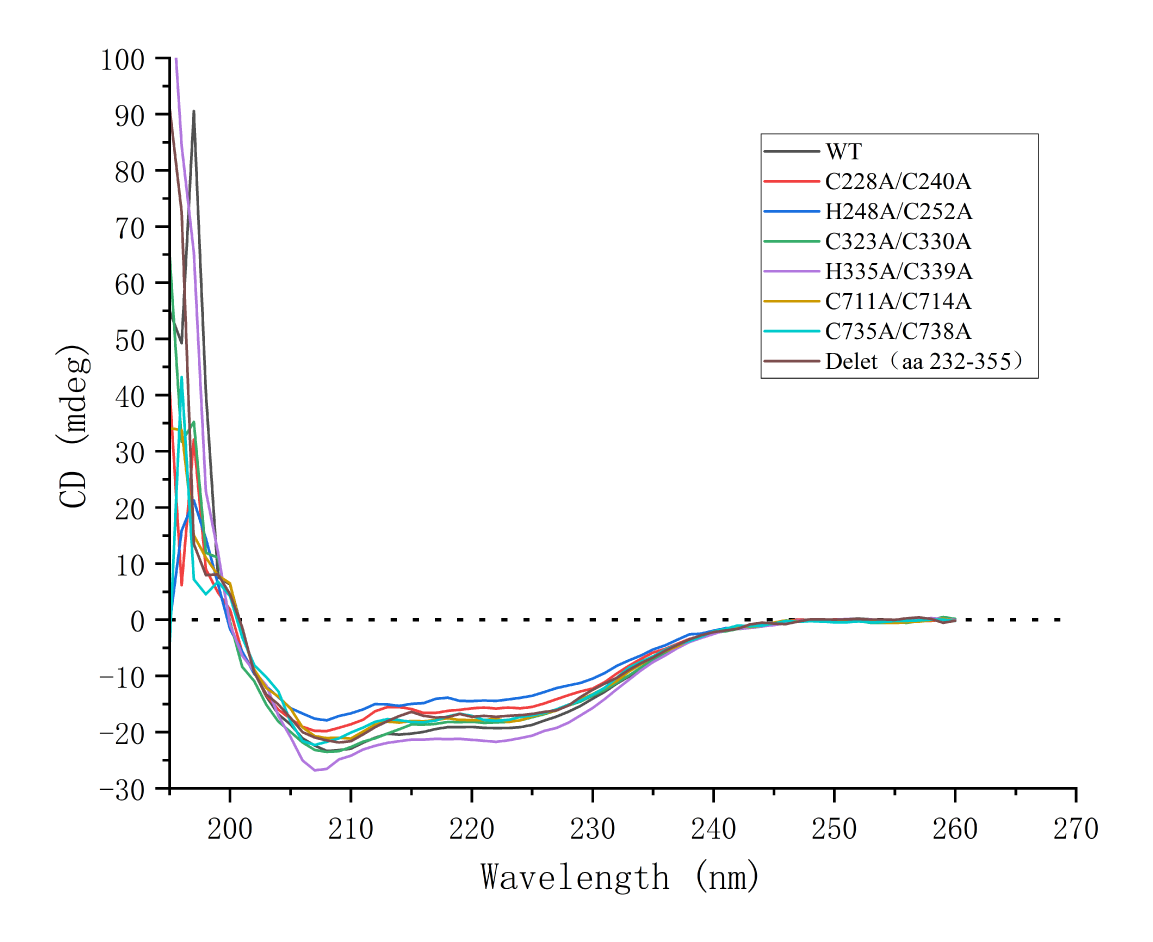

Supplement: S5 Fig — (TIF) [file pgen.1010930.s005.tif]

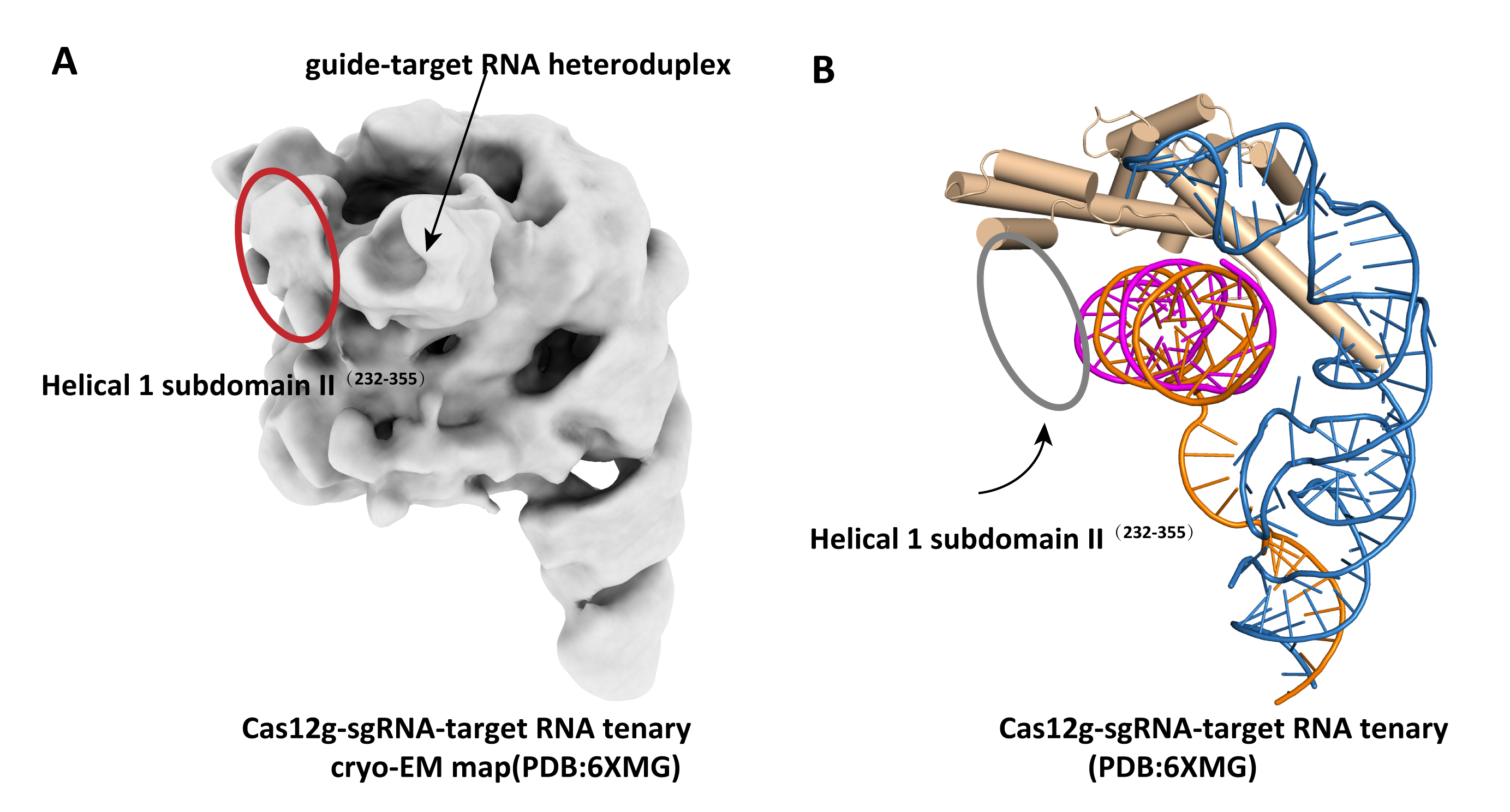

Supplement: S6 Fig — (A) The cryo-EM map of the Cas12g-sgRNA-target RNA complex (PDB code: 6XMG). Helical 1 subdomain II (aa 232–355) region is marked with a red box. (B) Structural representation of Helical 1 subdomain II (aa 232–355) region in Cas12g-sgRNA-target RNA complex (PDB code: 6XMG). (TIF) [file pgen.1010930.s006.tif]

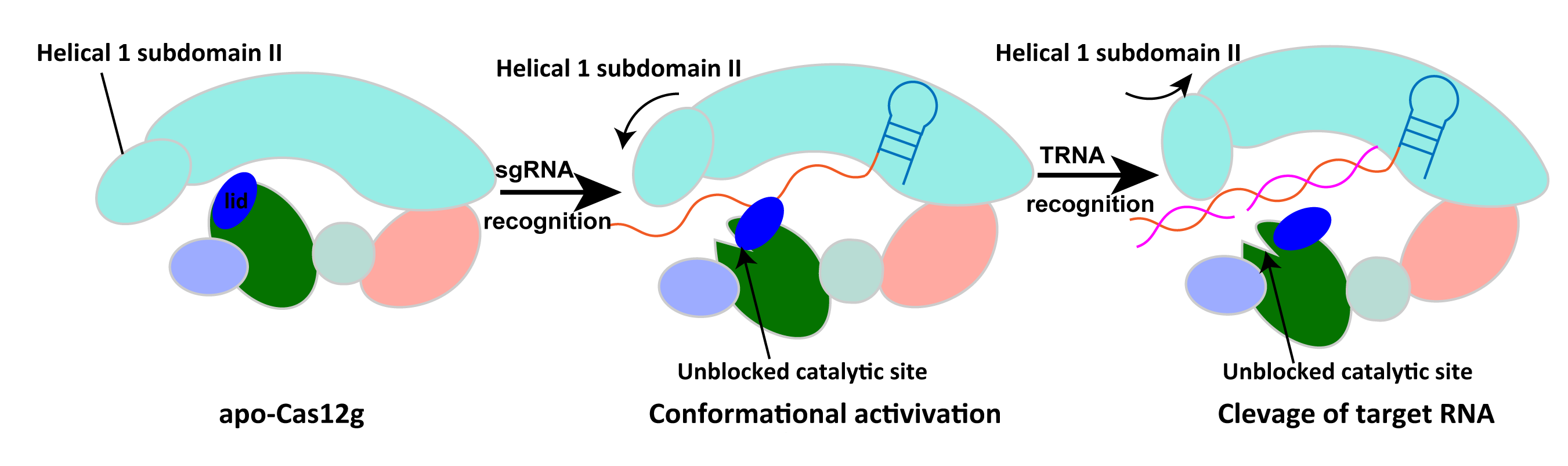

Supplement: S7 Fig — Each domain of Cas12g color coded as in Fig 1(A). (TIF) [file pgen.1010930.s007.tif]

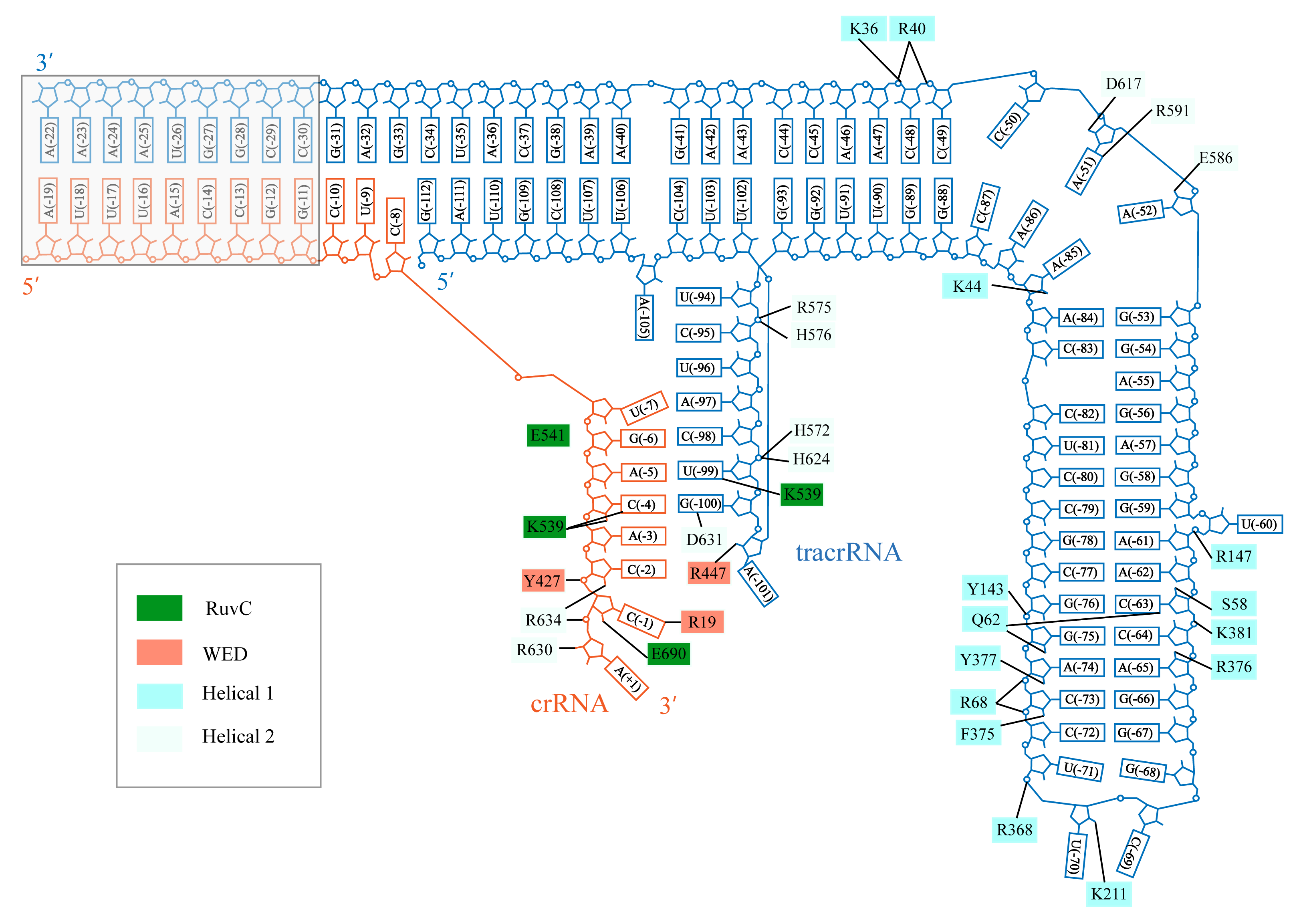

Supplement: S8 Fig — Domains and residues are colored according to Fig 1(A). (TIF) [file pgen.1010930.s008.tif]

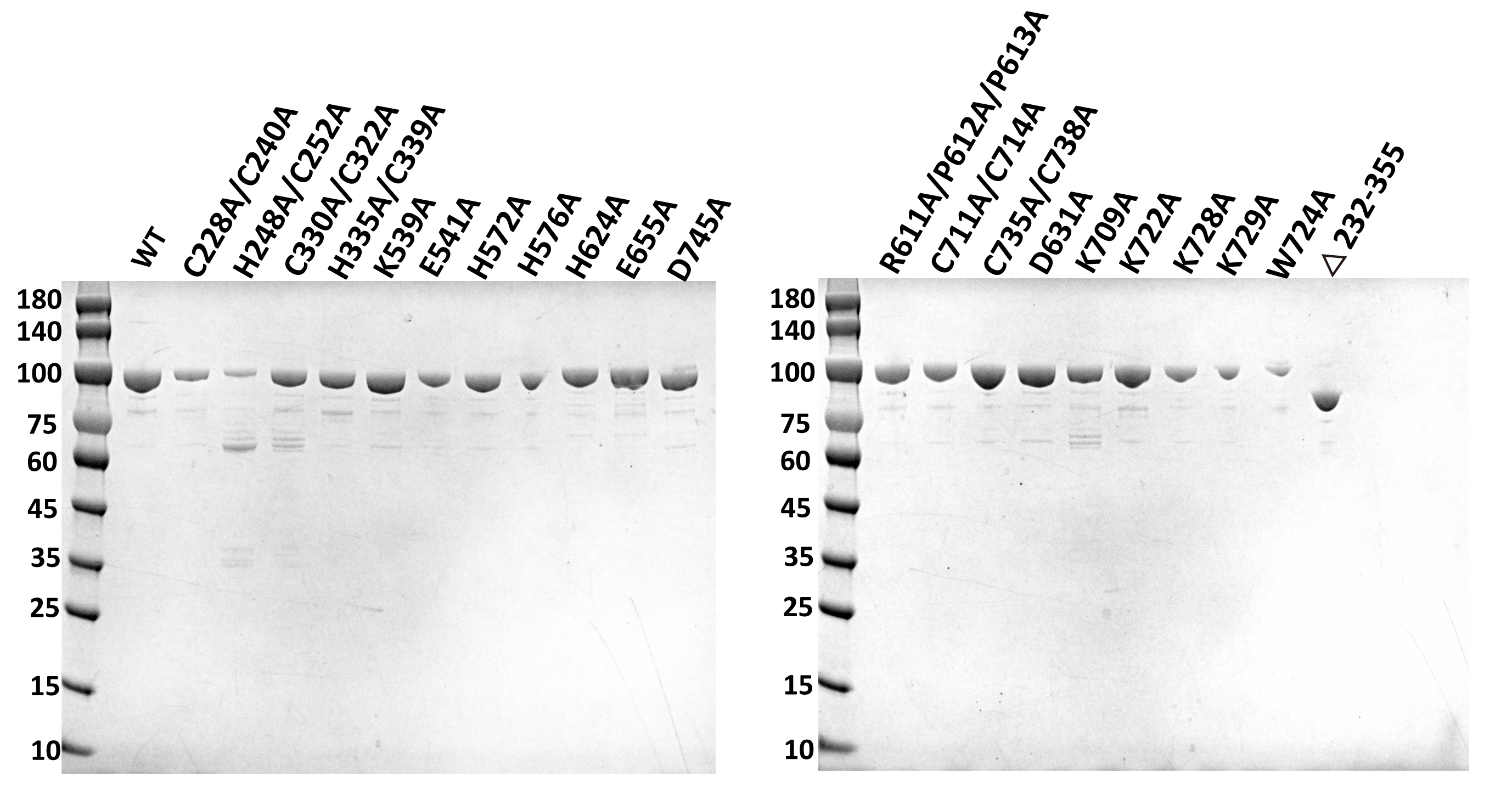

Supplement: S9 Fig — (TIF) [file pgen.1010930.s009.tif]
