# Supplementary material for: Structural transitions upon guide RNA binding and their importance in Cas12g-mediated RNA cleavage
Source: PLoS Genet. 2023 Sep 20;19(9):e1010930. doi: 10.1371/journal.pgen.1010930 (PMC10511118; doi:10.1371/journal.pgen.1010930)
Supplement: S1 Table — (DOCX) [file pgen.1010930.s010.docx]

**S1 Table. DNA coding sequence for Cas12g used in this study.**

| **Description** | **DNA coding sequence*** |
| --- | --- |
| Cas12g | ATGGCACAGGCGAGCAGCACCCCGGCGGTTTCGCCTCGTCCGCGTCCTCGTTACCGTGAAGAACGTACCCTGGTTCGTAAACTGCTGCCGCGTCCGGGGCAGAGCAAACAGGAATTTCGTGAAAATGTTAAAAAGCTGCGTAAAGCATTCCTGCAATTCAATGCAGATGTCAGCGGGGTGTGTCAGTGGGCAATTCAATTCCGTCCTCGTTATGGTAAACCGGCAGAACCGACCGAAACCTTTTGGAAATTCTTTCTGGAACCGGAGACCTCACTGCCGCCGAATGATAGCCGTAGCCCGGAATTTCGTCGCCTGCAGGCATTCGAAGCAGCAGCAGGTATTAATGGTGCAGCAGCACTGGATGATCCGGCCTTTACCAATGAACTGCGTGATAGCATTCTGGCAGTTGCAAGCCGTCCGAAAACCAAAGAAGCACAACGTCTGTTTAGTCGTCTGAAAGATTATCAACCGGCACATCGTATGATTCTGGCCAAAGTGGCGGCCGAATGGATTGAGTCTCGTTATCGTCGTGCACATCAAAATTGGGAACGTAACTATGAAGAATGGAAAAAGGAAAAACAGGAATGGGAACAAAATCACCCGGAACTGACCCCGGAAATTCGTGAAGCGTTTAACCAGATTTTTCAACAGCTGGAAGTGAAAGAAAAACGTGTTCGTATTTGTCCGGCAGCACGCCTGCTGCAGAATAAAGACAATTGCCAATATGCAGGTAAAAACAAACATAGTGTTCTGTGTAACCAGTTCAATGAATTTAAAAAGAACCACCTGCAGGGTAAAGCCATTAAATTTTTCTACAAAGACGCAGAAAAATACCTGCGCTGCGGTCTGCAGAGCCTGAAACCAAATGTTCAGGGACCATTCCGCGAAGATTGGAACAAATACCTGCGTTATATGAATCTGAAAGAAGAGACCCTGCGTGGTAAAAACGGGGGTCGTCTGCCGCATTGTAAAAATCTGGGTCAGGAGTGTGAATTTAATCCTCATACCGCCCTGTGTAAACAGTATCAGCAGCAGCTGAGCAGCCGTCCAGATCTGGTTCAGCATGATGAACTGTATCGTAAATGGCGCCGCGAATATTGGCGTGAGCCGCGTAAACCGGTTTTCCGTTATCCGAGCGTTAAACGTCATAGCATTGCAAAAATTTTCGGTGAAAATTACTTCCAGGCAGATTTTAAAAACAGCGTTGTTGGACTGCGTCTGGATTCTATGCCGGCAGGTCAGTATCTGGAATTTGCATTCGCACCATGGCCGCGTAATTACCGTCCGCAGCCGGGTGAGACCGAAATTAGCAGCGTGCATCTGCATTTTGTTGGTACCCGTCCGCGTATCGGTTTTCGTTTTCGTGTTCCGCATAAACGTAGCCGTTTTGATTGTACCCAGGAAGAACTGGATGAGCTGCGTAGCCGTACATTTCCTCGTAAAGCCCAGGATCAGAAATTTCTGGAGGCAGCCCGTAAACGTCTGCTGGAAACCTTTCCGGGTAATGCAGAACAGGAACTGCGTCTGCTGGCAGTGGCACTGGGCACCGATTCAGCACGTGCAGCATTTTTTATTGGTAAAACCTTTCAGCAGGCATTTCCTCTGAAAATTGTGAAAATTGAAAAACTGTACGAGCAATGGCCGAATCAGAAACAGGCTGGTGATCGTCGTGATGCAAGTAGTAAACAGCCGCGTCCGGGCCTGAGCCGTGATCACGTTGGTCGCCACCTGCAGAAAATGCGTGCACAGGCAAGCGAAATTGCACAGAAACGTCAGGAACTGACCGGCACCCCGGCGCCAGAAACCACCACCGATCAGGCAGCAAAAAAAGCAACCCTGCAGCCGTTTGATCTGCGTGGTCTGACCGTTCATACCGCACGTATGATTCGTGATTGGGCACGTCTGAATGCACGTCAGATTATTCAGCTGGCAGAAGAAAATCAGGTTGATCTGATTGTTCTGGAAAGCCTGCGTGGTTTTCGTCCGCCGGGTTATGAAAATCTGGATCAGGAAAAAAAACGTCGTGTTGCATTTTTTGCACATGGTCGTATTCGTCGTAAAGTTACCGAAAAAGCAGTTGAACGTGGTATGCGTGTTGTTACCGTTCCGTATCTGGCAAGCAGCAAAGTTTGTGCAGAATGTCGTAAAAAACAGAAAGATAATAAACAGTGGGAAAAAAATAAAAAACGTGGTCTGTTTAAATGTGAAGGTTGTGGTAGCCAGGCACAGGTTGATGAAAATGCAGCACGTGTTCTGGGTCGTGTTTTTTGGGGTGAAATTGAACTGCCGACCGCAATTCCGTAA |

* Codons have been optimized for expression in E. coli Rosetta (DE3) cells.
