# Supplementary material for: Structural transitions upon guide RNA binding and their importance in Cas12g-mediated RNA cleavage
Source: PLoS Genet. 2023 Sep 20;19(9):e1010930. doi: 10.1371/journal.pgen.1010930 (PMC10511118; doi:10.1371/journal.pgen.1010930)
Supplement: S2 Table — (DOCX) [file pgen.1010930.s011.docx]

**S2 Table. RNA transcription template used in this study.**

| **Description** | **RNA coding sequence*** |
| --- | --- |
| Cas12g-sgRNA | GGGATGCTTACTTAGTCATCTGGTTGGCAAACCTCCGCGGACCTTCGGGACCAATGGAGAGGAACCCAGCCGAGAAGCATCGAGCCGGTAAATGAATTTACCGGCTCTGACACCAATTCGAAATTAACACAAACAAGCT |
| **Cas12g-target RNA** | UCGAUAAGGUUGCUUGUUUGUGUUAAUUUCGAAUUAAGCU |
