# Supplementary material for: Structural transitions upon guide RNA binding and their importance in Cas12g-mediated RNA cleavage
Source: PLoS Genet. 2023 Sep 20;19(9):e1010930. doi: 10.1371/journal.pgen.1010930 (PMC10511118; doi:10.1371/journal.pgen.1010930)
Supplement: S3 Table — (DOCX) [file pgen.1010930.s012.docx]

**S3 Table. X-ray crystallography data collection and refinement statistics.**

| **Dataset** | **SeMet-Cas12g** |
| --- | --- |
| **Data collection** |  |
| Beamline | BL-17U1，SSRF |
| Wavelength (Å) | 0.9792 |
| Space group | *P*2_1_2_1_2_1_ |
| Cell dimensions |  |
| a, b, c (Å) | 64.07, 95.12, 167.51 |
| α, β, γ (°) | 90，90，90 |
| Resolution range (Å)* | 62.86-2.24 (2.30-2.24) |
| Completeness (%) | 99.4 (98.2) |
| I/σ(I) | 17.0 (2.3) |
| CC(1/2) | 0.997 (0.786) |
| *R*_merge_ | 0.091 (1.059) |
| Multiplicity | 13.0 (11.2) |
| **Refinement** |  |
| Resolution (Å) | 2.24 |
| No. unique reflections | 49819 |
| Rwork/ Rfree (%) | 22.4/26.8 |
| No. atoms |  |
| Protein | 5338 |
| Zn^2+^ | 2 |
| Solvent | 42 |
| B-factors (Å^2^) |  |
| Protein | 67.70 |
| Zn^2+^ | 71.99 |
| Solvent | 48.92 |
| R.m.s deviations | |
| Bond length (Å) | 0.009 |
| Bond angles (°) | 1.665 |
| Ramachandran plot (%) |  |
| Favored region | 96.52 |
| Allowed region | 3.48 |
| Outliers region | 0.00 |

*Highest resolution shell is shown in parentheses.
