# Supplementary material for: Structural transitions upon guide RNA binding and their importance in Cas12g-mediated RNA cleavage
Source: PLoS Genet. 2023 Sep 20;19(9):e1010930. doi: 10.1371/journal.pgen.1010930 (PMC10511118; doi:10.1371/journal.pgen.1010930)
Supplement: S4 Table — (DOCX) [file pgen.1010930.s013.docx]

**S4 Table. Statistics of cryo-EM data of the Cas12g binary complex and structure refinement.**

| **Data collection and processing** | |
| --- | --- |
| magnification | 165,000 |
| Voltage (kV) | 300 |
| electron exposure(e−/Å^2^) | 50 |
| Defocus range (μm) | 1.2- 1.8 |
| Pixel size (Å) | 0.84 |
| Symmetry imposed | C1 |
| Initial particle images (no.) | 570,000 |
| Final particle images (no.) | 88,696 |
| map resolution (Å) | 3.1 |
| FSC threshold | 0.143 |
| map resolution range (Å) | 2.8-20 |
| map sharpening *B* factor (Å^2^) | -50 |
| **model composition** | |
| Protein residues | 600 |
| Nucleotides | 90 |
| ***B* factors (Å**^2^**)** | |
| Protein | 25.24/112.35/59.54 |
| Nucleic acids | 42.38/208.07/91.04 |
| Zn^2+^ | 102.95/102.95/102.95 |
| **R.m.s. deviations** | |
| Bond lengths (Å) | 0.004 |
| Bonds angles (°) | 0.791 |
| **Validation** | |
| molProbity score | 2.56 |
| Clash score | 11.63 |
| Ramachandran plot |  |
| Favored (%) | 92.4 |
| Allowed (%) | 7.43 |
| Outliers region (%) | 0 |
